# Supplementary material for: Reversible metal cluster formation on Nitrogen-doped carbon controlling electrocatalyst particle size with subnanometer accuracy
Source: Nat Commun. 2024 Jul 20;15:6111. doi: 10.1038/s41467-024-50379-w (PMC11271611; doi:10.1038/s41467-024-50379-w)
Supplement: Supplementary file 1 — Supplementary Information [file 41467_2024_50379_MOESM1_ESM.pdf]

# Reversible Metal Cluster Formation on Nitrogen-doped Carbon Controlling Electrocatalyst Particle Sizes with Subnanometer Accuracy

*Janis Timoshenko*[1], Clara Rettenmaier[1], Dorottya Hursán[1], Martina Rüscher[1], Eduardo Ortega[1], Antonia Herzog[1], Timon Wagner[1], Arno Bergmann[1], Uta Hejral[1], Aram Yoon[1], Andrea Martini[1], Eric Liberra[1], Mariana Cecilio de Oliveira Monteiro[1], Beatriz Roldan Cuenya*[1]*

[1]Department of Interface Science, Fritz-Haber Institute of the Max-Planck Society, 14195 Berlin, Germany

[*] Corresponding author. E-mail: janis@fhi-berlin.mpg.de; roldan@fhi-berlin.mpg.de

## Supplementary Notes

**Supplementary Note 1. Evolution of copper clusters under static reaction conditions**

The reversible formation of copper particles in copper and nitrogen co-doped carbon (Cu-N-C) materials under static $CO_2$ reduction reaction ($CO_2RR$) conditions was previously observed by us using *operando* X-ray absorption fine structure (XAFS) method (conventional XAFS measurements with time resolution of ca. a few minutes per spectrum).[1,2] Nonetheless, no information was thus far available about the transformations taking place under pulsed $CO_2RR$. Our new set of quick XAFS (QXAFS) data with a time resolution up to 2 s per spectrum allows a more detailed insight into the restructuring of Cu-N-C catalysts not only under pulsed $CO_2RR$, which is the main goal of our current work, but also under static $CO_2RR$ conditions. Figure 1 in the main text shows the representative XANES and EXAFS spectra collected under static $CO_2RR$ (Figure 1ab). Corresponding raw EXAFS data in *k*-space are shown in Supplementary Figure 2. All static $CO_2RR$ experiments were performed at -1.35 V with respect to the reversible hydrogen electrode (RHE). The results obtained under static conditions are helpful for the understanding of the more complex evolution of the catalyst under pulsed reaction conditions.

In agreement with our previous report,[2] the analysis of X-ray absorption near edge structure (XANES) and extended X-ray absorption fine structure (EXAFS) features suggests that in the as-prepared Cu-N-Cs the Cu species are present in a $Cu^{2+}$ state with a distorted octahedral coordination. This corresponds to a planar Cu-$N_4$ unit, with two axial O or OH groups attached to the Cu center.[1,2] In particular, XANES data for the as-prepared sample exhibit an intense white line feature at ca. 8990 eV (Figure 1a in the main text), while the Fourier-transformed (FT) EXAFS spectrum is dominated by a single peak at ca 1.4 Å (phase uncorrected), corresponding to the bonding with low-Z elements (C, O or N) (Figure 1b in the main text). EXAFS data fitting (Figure 1b in the main text, Supplementary Figures 3-4, Supplementary Table 1) suggests that singly dispersed Cu species are six-coordinated, with a bond length (ca. 1.92 Å) slightly shorter than the Cu-O bond length in CuO or $Cu(OH)_2$ (ca. 1.94 Å) (Supplementary Figure 4), and a relatively large bond length disorder factor $\sigma^2$ (Supplementary Figure 4e). Lack of contributions of more distant coordination shells in FT-EXAFS spectra for the as-prepared samples is in agreement with the expected singly dispersed nature of $Cu^{2+}$ sites.

Under applied static potential XANES and EXAFS features change rapidly, and already after one minute under $CO_2$RR conditions, XANES and EXAFS spectra for our Cu-N-C catalyst strongly resemble those of the Cu foil, albeit with a lower amplitude of all main features (Figure 1ab in the main text). In particular, in EXAFS spectra we observe a gradual decrease of the peak at 1.4 Å, and instead a new peak at ca. 2.2 Å arises corresponding to the formation of Cu-Cu bonds in metallic clusters. Furthermore, additional FT-EXAFS peaks appear in the *R*-range between 3 and 6 Å, matching well those expected for fcc-type metallic particles.

For the quantitative analysis of these structural and chemical changes we use both XANES and EXAFS spectra. We interpret the changes in XANES using a linear combination analysis (LCA), where experimental spectra recorded at different time moments after the onset of the $CO_2$RR are expressed as a linear combination of spectra for the Cu foil and for the as-prepared Cu-N-C. The weights of these reference spectra in the linear combination correspond to the relative concentrations of metallic Cu and Cu remaining in the form of singly dispersed sites. Representative examples of LCA-XANES fits are shown in Supplementary Figure 5. On the other hand, concentrations of singly dispersed sites $x_{SAC}$ can also be deduced from EXAFS fits (from Cu-N coordination numbers, see Methods section in the main text). The $x_{SAC}$ values extracted from XANES and EXAFS are in a good agreement (Supplementary Figure 6). They suggest that under static $CO_2$RR, the concentration of singly dispersed Cu sites drops quickly (within the first 100 s) to ca. 20-30% (Figure 2a in the main text, Supplementary Figure 6a). Upon a more detailed look (Supplementary Figure 7), the time-dependency of the concentration of singly dispersed sites appears to differ from a simple exponential decay, and the quick initial drop in the concentration of single sites is followed by a much slower further decrease during the next few hours, until the concentration of these sites stabilizes at a value of ca. 12%.

The singly dispersed Cu sites observed under $CO_2$RR conditions exhibit similar distances to the nearest neighbor (N, C or O) in the as-prepared samples. This does not necessarily indicate that the local structure of these sites remains the same. In fact, as discussed in our previous work, we expect the axial O atoms to be replaced by CO adsorbates under $CO_2$RR conditions, thus, these species might play a role in $CO_2$RR mechanisms.[2] However, the effect of these possible rearrangements on XAFS data seem to be small in the case of Cu-N-C (unlike it is for Ni-N-C[1]). Here this observation plays in our favor and justifies our method for the XANES spectra

interpretation: when performing LCA-XANES, we can use the XANES spectrum for the as-prepared Cu-N-C as a reference for singly dispersed Cu species under working conditions.

For the purposes of our current work, however, the most interesting is the local structure of Cu species that do form metallic clusters. Knowing the concentrations of these species from LCA-XANES or from EXAFS fitting, and knowing the apparent Cu-Cu coordination numbers (averaged over all species in the sample) as obtained in EXAFS fit, we can estimate the true coordination numbers (corrected for the presence of metallic and non-metallic species) and, consequently, the sizes of the formed Cu particles. For the latter we can use, e.g., a simple relationship between the particle size and the coordination number proposed by Calvin et al (Figure 2f in the main text, inset).[3] The final true Cu-Cu coordination number observed in static $CO_2$RR experiment, 8.5±0.1 (after the correction for the coexistence of different species), corresponds to particles with diameter of ca. 1.3±0.1 nm (Figure 2f in the main text, inset).[3] While this relationship between coordination number and particle size is just an approximation, and we assume here that all particles in the sample have the same size and spherical shape, the obtained value is a convenient estimation of the effective size of Cu clusters formed under static $CO_2$RR conditions.

Benefiting from the high temporal resolution of our measurements, we can also track how fast the sizes of the Cu clusters increase (Figure 2d in the main text). We observe that under the static $CO_2$RR, the true Cu-Cu coordination number grows steadily from 0 to the aforementioned value of 8.5. Rather surprisingly, the final size of the Cu clusters is reached very quickly, within the first 100 s (Figure 2d in the main text). The characteristic time of Cu cluster growth thus is similar to the characteristic time of the conversion of singly dispersed Cu sites into metallic species. No further changes in the Cu-Cu coordination numbers and cluster sizes are observed under stationary $CO_2$RR conditions. One can also note that the increase of Cu cluster size is paralleled by an increase in Cu-Cu distance from ca. 2.52 Å shortly after the onset of $CO_2$RR, to ca. 2.56 Å after 10 min under working conditions (Supplementary Figure 4a). This trend is an agreement with the fact that for Cu clusters of subnanometer size somewhat compressed interatomic distances are expected.[4] The analysis of Cu-metal interatomic distances also allows us to rule out alloying of Cu species with Zn species remaining in the Cu-N-C catalyst after synthesis from ZIF-8 precursor. Indeed, in the latter case the Cu-metal distances are expected to be noticeably longer than in pure metallic Cu.[5,6]

Furthermore, the disorder factor $\sigma^2$ for the Cu-Cu bond increases during the first 100 s under $CO_2$RR, and then decreases again by ca. 0.005 Å in the next 100 s, reaching its final value of ca 0.011 Å (Supplementary Figure 4b). The latter is comparable with that of bulk Cu at room temperature. This could be interpreted as an evidence of the changes in the distribution of particle sizes: the population of uniformly ultrasmall particles formed immediately after the onset of $CO_2$RR, is first transformed into an ensemble of particles with sizes between 0 and 1.3±0.1 nm. The different particle sizes result also in the coexistence of different Cu-Cu bond lengths and an increase in the $\sigma^2$ value (increase in static disorder). The following decrease of the $\sigma^2$ value, in turn, suggests that the particle size distribution becomes narrower again, as the ultrasmall particles become larger. Indeed, the expected difference in $\sigma^2$ factors for a sample featuring identical monodisperse particles with the same Cu-Cu bond-length $R_1$ = 2.52 Å (or $R_2$ = 2.56 Å), and a sample containing 1:1 mixture of particles with bond lengths $R_1$ and $R_2$, respectively, is $0.25(R_2 - R_1)^2$ = 0.004 Å$^2$, which agrees well with the actual changes in $\sigma^2$ values observed in our static $CO_2$RR experiment.

Finally, we address the question of the stability of Cu species toward dissolution. By tracking the intensity of the Cu fluorescence signal, we can investigate the relative changes in Cu concentration in the sample (Supplementary Figure 8). As suggested by the obtained results, while under applied static potential there is indeed some loss in Cu fluorescence intensity, only ca. 20% of Cu is lost during the 5000 s of our static experiment. We mention here that also for our pulsed $CO_2$RR experiments, discussed in the main text, the degree of sample dissolution was similarly low (Supplementary Figure 8). This stability of the Cu species can be explained by the strong interactions of the Cu species with the N-C support, which also ensures the reversibility of Cu cluster formation, and prevents the metallic particles from unlimited agglomeration. We acknowledge, nonetheless that the demonstrated stability of our catalyst on the time scale of 5000 s does not guarantee its stability on longer time scales relevant for practical application of this catalytic system, and that this deserves separate future investigations.

**Supplementary Note 2: Principal Component Analysis**

Before we can rely on linear combination analysis (LCA) of XANES spectra for the interpretation of the changes in catalyst structure, we must first justify that for LCA-XANES under pulsed $CO_2$RR the same two reference spectra are sufficient as used for static $CO_2$RR conditions, namely, the spectrum for a metallic foil and for the as-prepared Cu-N-C catalyst. Indeed, the inclusion of additional standards, such as reference spectra for Cu(I) and Cu(II) oxides, in the LCA does not improve the quality of the LCA-XANES fits. Nonetheless, to confirm that only two spectroscopically distinct species are present in our catalysts under pulsed $CO_2$RR, we relied on principal component analysis (PCA) of XANES spectra collected under pulsed $CO_2$RR.

The objective of PCA is to express the dataset containing all the experimental spectra as linear combinations of as few as possible linearly independent vectors (principal components (PCs)). The number of the PCs, required to explain the variations between all the experimental spectra defines the dimensionality of the dataset, and is thus linked to the number of unique species contributing to the collected experimental data.[7,8] Here PCA was carried out for the dataset containing the experimental spectra collected under the pulsed $CO_2$RR with $E_c$ = -1.35 V, $E_a$ = 0.44 V and $\Delta t_a$ = $\Delta t_c$ = 30 s. Before the PCA procedure, from each spectrum we subtracted the average spectrum, averaged over the entire dataset. As is clear from Supplementary Figure 12a, the combination of the average spectrum with just a single PC is sufficient to reasonably describe all the experimental spectra. Furthermore, the features of the 1$^{st}$ PC have a clear interpretation (Supplementary Figure 12b): namely, a more positive weight of the 1$^{st}$ PC increases the intensity of the XANES shoulder feature, and decreases the intensity of the main XANES feature (so called “white line”), thus making the spectrum more similar to the spectrum of metallic Cu. The 2$^{nd}$ and higher PCs, in turn, do not contain unique spectroscopic features, and rather change the shape of the existing features. As a result, inclusion of additional PCs results only in an incremental decrease of the spectra approximation error (Supplementary Figure 12c). The 2$^{nd}$ and higher PCs thus account for the gradual changes in the structure of existing species (and experimental noise), rather than corresponding to an additional unique structural motif. The evolution of the weight of the first PC (Supplementary Figure 12d) tracks the changes in the fraction of metallic Cu species under pulsed $CO_2$RR, and is in a good agreement with the results of the LCA of XANES spectra (Figure 2b in the main text).

PCA allows us to conclude that indeed only two different species are present in the dataset, one corresponding to metallic Cu, and the other corresponding to cationic Cu species that from XAS perspective are indistinguishable from the cationic Cu present in the as-prepared Cu-N-C catalysts. Based on EXAFS data analysis and the results of ex-situ characterization of the as-prepared samples,[2] these cationic species are attributed to singly-dispersed Cu sites. The lack of additional contributions in the PCA suggests strongly that no additional species (e.g., oxide particles or Cu(I) species) appear under pulsed conditions in detectable amounts. We also note that the formation of Cu(I) oxide species (the most likely oxide species under $E_a$ = 0.44 $V_{RHE}$[9]) would result in noticeably shorter Cu-O distances (ca. 1.836 Å)[9] than those observed in the fitting of the EXAFS data collected for Cu-N-C catalysts under pulsed reaction conditions.

Taken together, EXAFS, LCA and PCA XANES results thus point toward a non-trivial conclusion that the exposure of metallic Cu clusters to anodic potentials and their transformation into singly dispersed Cu sites is not associated with the formation of intermediate species such as copper oxides.

**Supplementary Note 3: Formation of metallic clusters during cathodic potential pulse**

Figure 2 and Figure 3 in the main text show an interesting trend that the changes in the concentration of singly dispersed Cu sites ($x_{SAC}$ value) during the potential pulses are linked to the concentration of these species themselves: the larger is the $x_{SAC}$ value, the larger is the amplitude of the oscillations. This is true both, for the first few potential cycles, where the catalyst is dominated by the singly dispersed species (Figure 2 in the main text), as well as for the stationary state that is reached after many potential cycles (Figure 3 in the main text). In fact, the amount of singly dispersed Cu sites that is transformed into metallic species during each cathodic potential pulse ($\Delta x_{SAC}$) appears to be linearly correlated with the concentration of singly dispersed Cu sites at the onset of the respective cathodic pulse. This relationship is illustrated in the Supplementary Figure 13, and appears to be very robust. In fact, the results of three separate measurements with the same $\Delta t_c$ = 30 s value, but different $\Delta t_a$ values all follow the same linear trend. Note that the results for $\Delta t_a$ =$\Delta t_c$ = 30 s and for other $\Delta t_a$ values were obtained during different beamtimes at different synchrotrons. Similar relationships for other $\Delta t_c$ values are shown in Supplementary Figure 14.

The observed linear relationship in Supplementary Figure 13 has a simple explanation: it indicates that under the applied cathodic pulse of fixed length, the probability for a given remaining cationic single atom site to be converted into a metallic species is constant, regardless of the sample history and amounts of other species present in the sample. In this scenario, the number of cationic single sites that are converted into metal during each pulse is just proportional to the number of single cationic sites remaining in the sample.

One should note, however, that the linear trendlines in Supplementary Figure 13 and Supplementary Figure 14 do not go through the (0,0) point. Instead, $\Delta x_{SAC}$=0 level is crossed at certain finite $x_{SAC}$ value $x_0$, which for the $\Delta t_c$ = 30 s case is ca. 0.15. For $\Delta t_c$ = 16 s and $\Delta t_c$ = 8 s (Supplementary Figure 14ab), a very similar corresponding $x_0$ value is obtained (0.17 and 0.11, respectively). These results thus indicate that 11-17 % of single sites are not involved in these transformations, and will remain in their single-atom dispersed state. This correlates remarkably with our observation from the static $CO_2$RR experiment (Supplementary Figure 7) that ca. 12% of the singly dispersed Cu sites are not converted into the metallic state even after more than 1 h of static $CO_2$RR at -1.35 V.

On the other hand, for pulsed $CO_2$RR with shorter cathodic pulses ($\Delta t_c$ = 4 s and $\Delta t_c$ = 2s, Supplementary Figure 14cd), the $x_0$ value is larger (0.26 and 0.32, respectively). This, again, correlates with our findings for static $CO_2$RR, which showed that even among the singly dispersed species that do eventually get converted into metallic particles, there are orders of magnitude differences in terms of how fast this transformation occur (Supplementary Figure 7). Some of the species that have slower response, are converted to metallic state upon long cathodic pulses, but remain stable under short cathodic pulses.

If we now focus on the population of more dynamic species (i.e., those that eventually do get converted into their metallic state under given pulse conditions), we conclude that in the $\Delta t_c$ = 30s case, the probability for them to be converted into metallic species during each cathodic potential pulse is ca. 75% (given by the slope of the trendline in Supplementary Figure 13). One could expect that by reducing the length of the cathodic pulse, this probability would be suppressed strongly. However, our results shown in Supplementary Figure 14 indicate that this probability depends weakly and unsystematically on the $\Delta t_c$ value, and even at $\Delta t_c$ = 2s it still is similar (78%) to our observations for the $\Delta t_c$ = 30 s case. This suggests that the transformations of singly dispersed sites into metallic species can be very quick, faster than the duration of the shortest

cathodic pulse that we have tested (2 s). This confirms that the catalyst transformations under short cathodic pulses is dominated by the contribution of very dynamic single Cu species, while the more stable Cu single sites remain unaffected. In contrast, during the longer cathodic pulses, there is sufficient time to also get the more stable Cu sites transformed, resulting is the smoother concentration profiles observed, Figure 3 (main text).

The findings of our static and pulsed $CO_2$RR experiments thus all highlight that even among the singly dispersed sites within the same sample there are significant differences in terms of their stability. The fact that some single sites are not transformed into metallic state could be explained simply by the fact that they are electrochemically not accessible (e.g., buried within the C-N support). However, as mentioned in Supplementary Note 1, we cannot clearly distinguish by XAS between the single Cu sites in the as-prepared state and single Cu sites remaining under $CO_2$RR conditions. Therefore, we cannot exclude that these more stable species do, in fact, participate in the $CO_2$RR mechanisms.

**Supplementary Note 4: Model for the evolution of Cu species in Cu-N-C catalysts**

To capture the main trends in the evolution of the Cu-N-C catalyst, we consider a strongly simplified model where only four different Cu species coexist: (i) dynamic single site cationic Cu species (those that can get converted into metallic species), (ii) stable single site cationic Cu species (those that remain as single site cationic species), (iii) small metallic Cu clusters and (iv) large metallic Cu particles. The relative concentration of Cu atoms present in the form of each of these four species we denote as $x_{\mathrm{SAC,d}}(t)$, $x_0$, $x_{\mathrm{S}}(t)$ and $x_{\mathrm{L}}(t)$, respectively. The sizes of “small clusters” and “large particles” can be characterized by the corresponding average Cu-Cu coordination numbers $CN_{\mathrm{S}}$ and $CN_{\mathrm{L}}$, respectively. The Cu-Cu coordination number for single site cationic species is 0.

The concentration of Cu within the stable cationic species ($x_0$) does not change. In turn, some dynamic single site Cu species are transformed into small metallic clusters during each cathodic pulse, resulting in the decrease of $x_{\mathrm{SAC,d}}$ and an increase in $x_{\mathrm{S}}$. The small metallic clusters, if present, can be transformed into large metallic particles. We assume that the amount of cationic Cu that is converted into small clusters is given by $x_{\mathrm{SAC},d} w_{\mathrm{SAC \to S}}$, while the amount of Cu in the small clusters that is converted into large particles is given by $x_{\mathrm{S}} w_{\mathrm{S \to L}}$. During each anodic pulse, in turn, large particles can fragment into small metallic clusters, while the small metallic clusters can

transform into single site cationic species. We assume that the amount of Cu within the large particles that is converted into small clusters is given by $x_{\mathrm{L}} w_{\mathrm{L}\to\mathrm{S}}$, while the amount of Cu within the small clusters that is converted into single sites is given by $x_{\mathrm{S}} w_{\mathrm{S}\to\mathrm{SAC}}$. Here the terms $w_{\mathrm{SAC}\to\mathrm{S}}$, $w_{\mathrm{S}\to\mathrm{L}}$, $w_{\mathrm{L}\to\mathrm{S}}$ and $w_{\mathrm{S}\to\mathrm{SAC}}$ characterize the probability of the corresponding transformation. We furthermore assume that these terms do not change with time. Nonetheless, these terms, clearly depend on the pulse parameters (pulse durations and applied potentials).

The changes in the populations of the different species at the end of each potential cycle are then given by the following set of recursive equations:

$$\begin{cases} x_0(t+\Delta t_c) = x_0(t+\Delta t_c+\Delta t_a) = x_0 \\ x_{\mathrm{SAC,d}}(t+\Delta t_c) = x_{\mathrm{SAC,d}}(t)(1-w_{\mathrm{SAC}\to\mathrm{S}}) \\ x_{\mathrm{S}}(t+\Delta t_c) = x_{\mathrm{S}}(t)(1-w_{\mathrm{S}\to\mathrm{L}}) + x_{\mathrm{SAC,d}}(t) w_{\mathrm{SAC}\to\mathrm{S}} \\ x_{\mathrm{L}}(t+\Delta t_c) = \mathrm{x}_{\mathrm{L}}(t) + x_{\mathrm{S}}(t) w_{\mathrm{S}\to\mathrm{L}} \\ x_{\mathrm{SAC,d}}(t+\Delta t_c+\Delta t_a) = x_{\mathrm{SAC,d}}(t+\Delta t_c) + x_S(t+\Delta t_c) w_{\mathrm{S}\to\mathrm{SAC}} \\ x_{\mathrm{S}}(t+\Delta t_c+\Delta t_a) = x_{\mathrm{S}}(t+\Delta t_c)(1-w_{\mathrm{S}\to\mathrm{SAC}}) + x_L(t+\Delta t_c) w_{\mathrm{L}\to\mathrm{S}} \\ x_{\mathrm{L}}(t+\Delta t_c+\Delta t_a) = x_{\mathrm{L}}(t+\Delta t_c)(1-w_{\mathrm{L}\to\mathrm{S}}) \end{cases} \tag{1}$$

The initial conditions are $x_{\mathrm{SAC,d}}(0) = 1 - x_0$; $x_S(0) = x_L(0) = 0$.

The total concentrations of singly dispersed cationic Cu sites at any moment of time is given by $x_{\mathrm{SAC}}(t) = x_{\mathrm{SAC,d}}(t) + x_0$. The average Cu-Cu coordination numbers (corrected for the presence of singly dispersed cationic Cu species) at any time can be calculated as $\mathrm{CN} = \frac{x_{\mathrm{S}}(t)\mathrm{CN}_{\mathrm{S}} + x_{\mathrm{L}}(t)\mathrm{CN}_{\mathrm{S}}}{x_{\mathrm{S}}(t) + x_{\mathrm{L}}(t)}$.

If the parameters $w_{\mathrm{SAC}\to\mathrm{S}}$, $w_{\mathrm{S}\to\mathrm{L}}$, $w_{\mathrm{L}\to\mathrm{S}}$, $w_{\mathrm{S}\to\mathrm{SAC}}$, $x_0$, $\mathrm{CN}_{\mathrm{S}}$ and $\mathrm{CN}_{\mathrm{L}}$ are known, the aforementioned equations fully define the evolution of the populations of different Cu species, and provide the information on the total concentration of cationic Cu single sites, and the average Cu-Cu coordination number, that can be directly compared with the results of LCA-XANES and EXAFS data fitting.

For the case of potential pulses with $\Delta t_c = \Delta t_a = 30s$ it is convenient to estimate the values of these parameters from our observations for the first few potential pulses (Figure 2 in the main text) and for the stationary state (Figure 3 in the main text). In the stationary state, at each cathodic pulse the fraction of singly dispersed Cu sites decreases from ca. 0.193 by ca. 0.035, and then increases again by the same amount during the anodic pulse. Furthermore, the average coordination number *CN* changed from ca. 8.0 at the end of anodic pulse to ca. 9.5 at the end of the cathodic pulse. We also note that in the stationary state the amount of Cu converted from small clusters to larger particles during the cathodic pulse should be equal to the amount of Cu converted from large particles to small particles. Furthermore, from Figure 2b we note that during the first cathodic pulse the concentration of singly dispersed Cu sites drops from 1.0 to ca. 0.284, while during the first anodic pulse it increases to ca. 0.558. Finally, according to this model, after the very first cathodic potential pulse, there are only small Cu clusters present in the samples, and no large Cu particles. Consequently, $\mathrm{CN_S}$ can be estimated to be equal to the Cu-Cu coordination number after the first 30s, or, alternatively, from the particle sizes observed in the static $CO_2$RR experiment after 30 s. To facilitate the extrapolation of this model to different pulse durations, here we exploited the latter option, which for 30 s pulses gives $\mathrm{CN_S} = 4.4$. According to the model in the inset of Figure 2f in the main text,[3] this corresponds to a particle diameter of ca. 0.6 nm, and is slightly lower than the coordination number (5.5) for a cuboctahedral $Cu_{13}$ cluster.

Plugging this information into the system of equations above, we can solve if for $w_{\mathrm{SAC}\to\mathrm{S}}$, $w_{\mathrm{S}\to\mathrm{L}}$, $w_{\mathrm{L}\to\mathrm{S}}$, $w_{\mathrm{S}\to\mathrm{SAC}}$, $x_0$ and $\mathrm{CN_L}$. In particular, we obtain $\mathrm{CN_L}$=10.1, $x_0$= 0.151, $w_{\mathrm{SAC}\to\mathrm{S}} = 0.844$, $w_{\mathrm{S}\to\mathrm{L}} = 0.810$, $w_{\mathrm{L}\to\mathrm{S}} = 0.322$, $w_{\mathrm{S}\to\mathrm{SAC}} = 0.382$. As shown in Supplementary Figure 15, with these parameters this model is able to capture reasonably all the key features of the evolution of Cu-N-C catalysts under 30 s potential pulses, including changes in $x_{\mathrm{SAC}}(t)$ and CN during the first few potential pulses, gradual decrease in the amplitude of $x_{\mathrm{SAC}}(t)$ oscillations with time, behavior of $x_{\mathrm{SAC}}(t)$ and CN in the stationary state.

To extrapolate this model to other durations of cathodic and anodic pulses, we need to define the dependencies of $w_{\mathrm{SAC}\to\mathrm{S}}$, $w_{\mathrm{S}\to\mathrm{L}}$, $w_{\mathrm{L}\to\mathrm{S}}$, $w_{\mathrm{S}\to\mathrm{SAC}}$ on pulse parameters. Considering that for very short pulses we expect all of these values to drop to 0, while for very long pulses they should approach 1, the simplest approach is to model each of these terms as $w_i = 1 - \exp(-\Delta t/\tau_i)$, where $\Delta t$ is equal to $\Delta t_c$ for $w_{\mathrm{SAC}\to\mathrm{S}}$ and $w_{\mathrm{S}\to\mathrm{L}}$, and equal to $\Delta t_a$ for $w_{\mathrm{S}\to\mathrm{SAC}}$ and $w_{\mathrm{L}\to\mathrm{S}}$. The

characteristic times $\tau_i$ can be obtained from the known values of $w_{\mathrm{SAC}\to\mathrm{S}}$ , $w_{\mathrm{S}\to\mathrm{L}}$ , $w_{\mathrm{L}\to\mathrm{S}}$ , $w_{\mathrm{S}\to\mathrm{SAC}}$ for $\Delta t_c = \Delta t_a = 30s$. The obtained values are $\tau_{\mathrm{SAC}\to\mathrm{S}} = 16\ s$, $\tau_{\mathrm{S}\to\mathrm{L}} = 18\ s$, $\tau_{\mathrm{L}\to\mathrm{S}} = 77\ s$, $\tau_{\mathrm{S}\to\mathrm{SAC}} = 62\ s$, indicating that the transformations of cationic species into the small clusters and the growth of particles are significantly faster than the inverse processes. We further assume that the dependency of $CN_S$ on the duration of cathodic pulse is given by Figure 3d in the main text, i.e., the clusters that are formed from singly dispersed Cu species during the cathodic pulse of duration $\Delta t_c$, have the same sizes as clusters formed under the static $CO_2$RR after $t = \Delta t_c$. For example, for $\Delta t_c = 2$, the $CN_S$ value will be ca. 0.4, which indicates the presence of single metallic atoms with a few dimers. The value of $CN_L$, in turn, we assume to be independent on of the duration of potential pulses.

Under these assumptions, the model predicts the changes in the concentrations of singly dispersed cationic Cu species and in the average Cu-Cu coordination numbers that are in semi-quantitative agreement with experimental observations (Figure 3bc in the main text, Supplementary Figure 20). Discrepancies between the experimental results and our model stem mainly from our simplified (exponential) dependencies of the transition rates on pulse durations. As discussed in Supplementary Note 3, the relationships between the probabilities for, e.g., single cationic site to be converted to metal clusters can be much more intricate due to, in part, orders of magnitude large differences in the characteristic response times even among the single site species. Considering the simplicity of our model (which, among other assumptions, assumes that particles of only two different sizes are present), we feel that the inclusion of these details in the model is not warranted and will not make it physically more meaningful.

**Supplementary Note 5: Effect of the anodic pulse duration**

Unlike the pronounced effect of different $\Delta t_c$ values on the catalyst structure and composition, the changes in $\Delta t_a$ seem to affect the system to a lesser degree. In Supplementary Figures 16-17, we show the results of XANES and EXAFS data analysis for pulsed $CO_2$RR with $\Delta t_c = 30$ s and $\Delta t_a = 16$ s or 4 s. In both these cases, the oscillations in system parameters seem to be much weaker than for the $\Delta t_c = \Delta t_a = 30$ s case. Instead, the system with lower $\Delta t_a$ values quickly reaches stationary state, with the final values of $x_{SAC}$ and Cu-Cu coordination number resembling those obtained in $\Delta t_c = \Delta t_a = 30$ s.

This observation can be reproduced by our model, developed in Supplementary Note 4. Supplementary Figure 18 shows the predicted average concentrations of cationic Cu species, small Cu clusters and large Cu nanoparticles, as well as the average Cu-Cu coordination number as a function of $\Delta t_a$ and $\Delta t_c$. Furthermore, to further validate these results, we performed additional experimental *operando* XAFS measurements for the pulse durations $\Delta t_a = \Delta t_c = 1$s, $\Delta t_a = \Delta t_c = 4$s, $\Delta t_a = 1$s with $\Delta t_c = 4$s and $\Delta t_a = 4$s with $\Delta t_c = 1$s (Supplementary Figure 19). By comparing the average concentrations of cationic Cu and average Cu-Cu coordination numbers, extracted from experimental data, with the predictions of our model, we find that at these shorter values of the anodic pulse durations, the agreement between experiment and model is, in fact, even better than for $\Delta t_a = 30$ s (Supplementary Figure 20), giving us further confidence in the validity of the conclusions based on our model.

From Supplementary Figure 18, for all $\Delta t_a$ values, qualitatively the same dependency of the catalyst parameters on the $\Delta t_c$ value can be observed, namely, the dominance of singly dispersed cationic species at low $\Delta t_c$ values, dominance of large Cu clusters at large $\Delta t_c$ values, and a significant contribution of small Cu clusters at intermediate $\Delta t_c$ values (Supplementary Figure 18cd). With increasing $\Delta t_a$, the maximum of the contribution of small Cu clusters shifts toward higher $\Delta t_c$, and for each $\Delta t_c$ value it is observed at $\Delta t_c \approx \Delta t_a/4$. This relation we attribute to the fact that the characteristic times of the main processes taking place during the anodic pulse (conversion of larger particles into smaller ones, and the conversion of small clusters into cationic species, $\tau_{\mathrm{L}\to\mathrm{S}}$ and $\tau_{\mathrm{S}\to\mathrm{SAC}}$, respectively), both are roughly four times longer than those of the corresponding inverse processes taking place during the cathodic pulse (Supplementary Note 4).

**Supplementary Note 6: Effect of sample history**

While in this work we focused on the reversible structural transformations of Cu-N-C catalysts under pulsed $CO_2$RR and the reversible changes in their catalytic function, such reversible changes, without doubt, are paralleled by some irreversible changes, e.g., in the catalyst support and catalyst morphology. While such changes are mostly invisible in XAS, they can have an impact on the catalyst performance. One of the consequences of this is that care is needed when comparing the results obtained for samples with different history. In our XAS measurements, as well as in Figure 4b in the main text, to reduce the systematic errors due to differences between samples, results were collected for the same sample, starting from the shortest pulses and exposing it to air (XAS)

and in open circuit potential (selectivity measurements) for 30 min in-between different pulse conditions. In contrast, in Figure 4a, selectivity measurements were performed by using a fresh sample for each condition. While the trends in Figure 4a and Figure 4b are qualitatively the same, for $\boldsymbol{\Delta t_c}$ values between 10 s and 35 s (region (ii) in Figure 4c), the ratio between $H_2$ and CO is noticeably lower for the fresh samples than for the re-used samples (ca. 1.3 in the former case and ca. 2.0 in the latter case for $\boldsymbol{\Delta t_c}$=16 s). To demonstrate that this is indeed linked to the irreversible changes in the catalyst accumulated during the reusage of the sample, in Supplementary Figure 24 we start with a fresh sample and expose it again to a series of pulse conditions with $\boldsymbol{\Delta t_a}$=30 s and varied $\boldsymbol{\Delta t_c}$, but this time starting from a larger value of $\boldsymbol{\Delta t_c = 12\ s}$. In this case, in region (ii) we observe much lower selectivities for $H_2$ than when starting pulse sequence with $\boldsymbol{\Delta t_c}$ = 0.5 s, as in Figure 4b. Specifically, for $\boldsymbol{\Delta t_c}$=16 s we obtain the ratio between $H_2$ and CO selectivities ca. 1.08, which is much closer to what was observed for fresh samples. Furthermore, once we reached the largest $\boldsymbol{\Delta t_c}$=150 s value, in Supplementary Figure 24 we continued with the same sample, and gradually reduced $\boldsymbol{\Delta t_c}$, returning eventually to $\boldsymbol{\Delta t_c}$=16 s and lower pulse durations. In this case, the production of $H_2$ increased, and the ratio between $H_2$ and CO selectivities was ca. 2.06, in good agreement with Figure 4b. We note that such hysteresis effect was not observed for CO and $C_2H_4$. At the same time, we observe that the prolonged re-usage of the same sample, and exposure to pulses with longer $\boldsymbol{\Delta t_c}$ values before returning to intermediate and short $\boldsymbol{\Delta t_c}$ values, results in a decrease of the methane production. This suggests that the larger Cu particles (the main producers of CO and $C_2H_4$) are less susceptible to irreversible changes in the catalyst than singly dispersed cationic sites and ultrasmall Cu clusters.

## Supplementary Figures

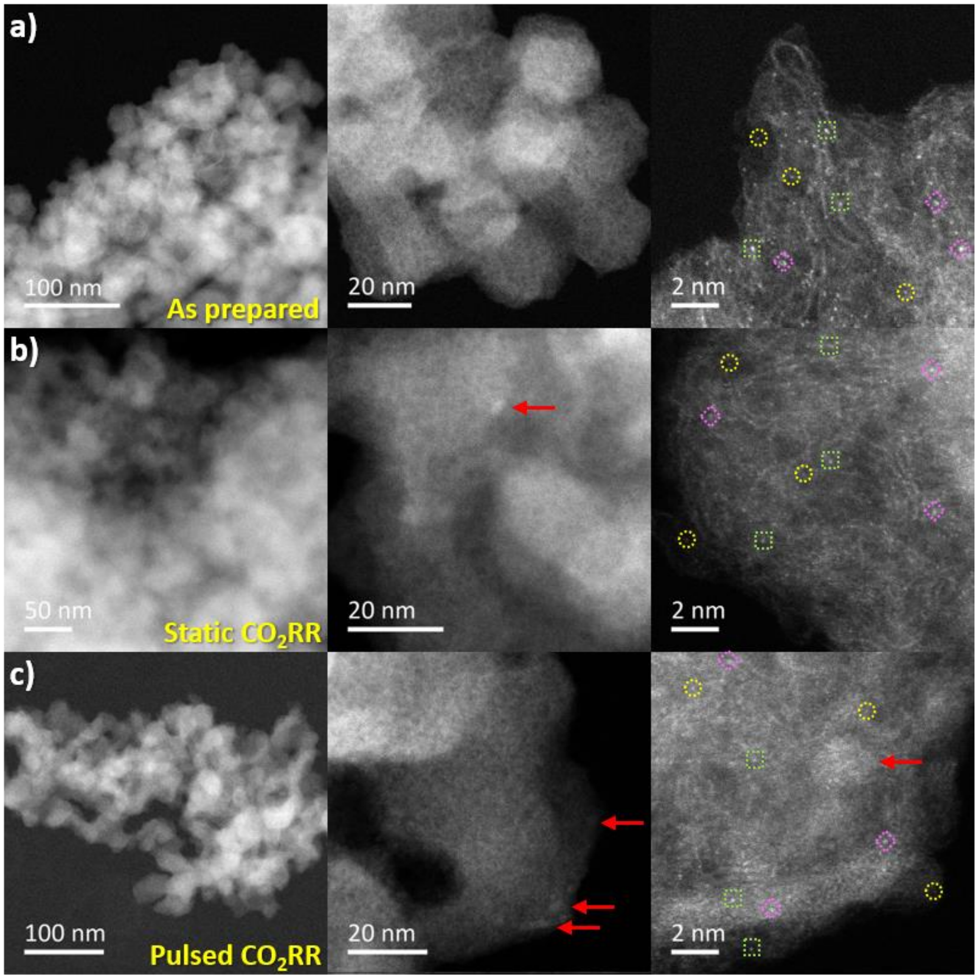


**Supplementary Figure 1. Ex-situ high angle annular dark-field scanning transmission electron microscopy (HAADF-STEM) images of the Cu-N-C electrocatalysts** in the as prepared state (a), after $CO_2RR$ at −1.35 V (b), and after pulsed $CO_2RR$ with 30 s long pulses of cathodic (-1.35 V) and anodic potential (+0.44 V) (c). High resolution HR-STEM images (right column) show the disordered structure of the carbon framework and highlight the presence of singly dispersed metal species (yellow circles), with some minor contribution of dimers (green squares), or larger ultradispersed structures (pink diamonds). In the as-prepared sample no nanoparticles can be detected. In both reacted samples, the presence of some nanoparticles and aggregates can be observed (marked with red arrows). These could be attributed to zinc oxide clusters (see Ref.[2]) although the presence of some residual Cu nanoparticles cannot be excluded (e.g., the particles that partially detached from the C-N support and thus could not redisperse, but, therefore, can also not contribute to the catalytic functionality). Considering the lack of a significant contribution from these species in our XAS data, only a small fraction of Cu can be involved in the formation of these species.

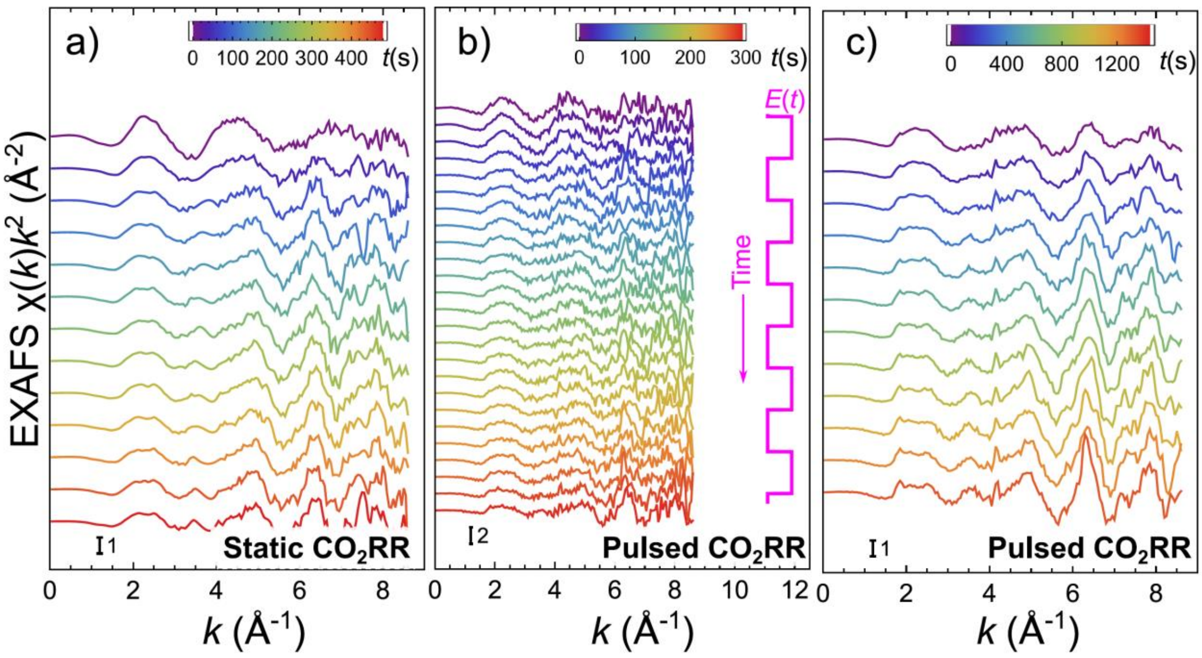


**Supplementary Figure 2.** ***Operando*** **Cu K-edge EXAFS spectra collected under static and pulsed $CO_2$RR.** Evolution of *operando* Cu K-edge EXAFS spectra for Cu-N-C catalysts during the first 400 s under static -1.35 V vs RHE (a), during the first 300 s under pulsed $CO_2$RR with $E_c$ = -1.35 V, $E_a$ = 0.44 V and $\Delta t_a = \Delta t_c$ = 30 s, and during the first 1400 s under these pulsed $CO_2$RR conditions. Depicted spectra are averages over 20 s (a), 6 s (b) or 60 s (c). Spectra are shifted vertically for clarity. $CO_2$-saturated 0.1 M $KHCO_3$ was used as an electrolyte.

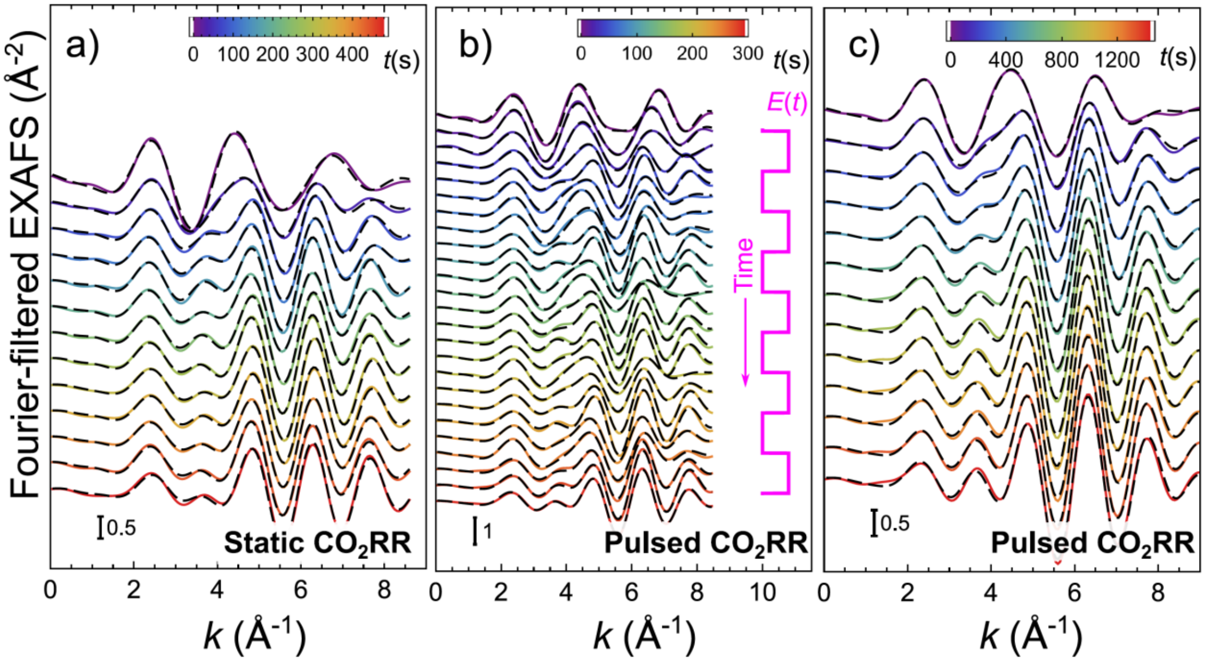


**Supplementary Figure 3**. **Fits of *operando* Cu K-edge EXAFS spectra collected under static and pulsed $CO_2$RR.** Comparison of experimental Fourier-filtered Cu K-edge EXAFS data (solid color lines) with fit results (black dashed lines). Results of EXAFS data fitting for Cu-N-C catalysts during the first 400 s under static -1.35 V potential (a), during the first 300 s under pulsed $CO_2$RR with $E_c$ = -1.35 V, $E_a$ = 0.44 V and $\Delta t_a = \Delta t_c$ = 30 s, and during the first 1400 s under these pulsed $CO_2$RR conditions are shown. The fitted spectra are averages over 20 s (a), 6 s (b) or 60 s (c). Spectra are shifted vertically for clarity.

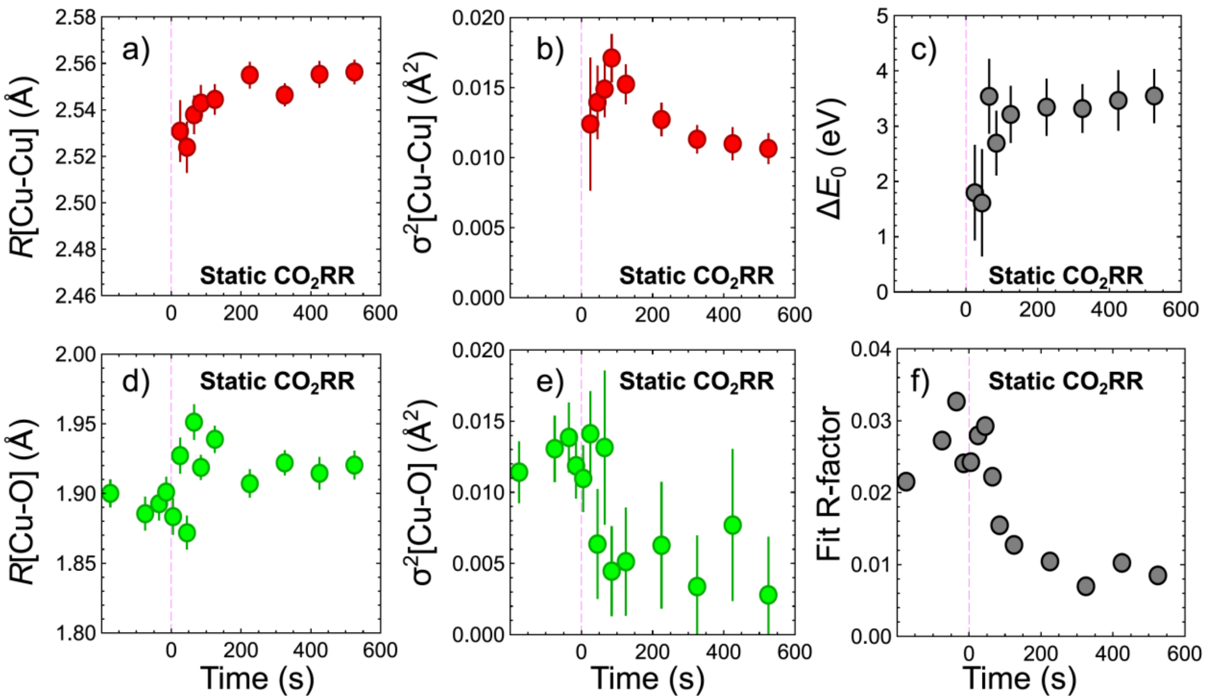


**Supplementary Figure 4. EXAFS data fitting results for the Cu-N-C catalysts under static -1.35 V potential.** The obtained values for Cu-Cu bond length (*R*) and bond length disorder ($\sigma^2$) are shown in (a,b). The obtained values for the Cu-O bond length and bond length disorder are shown in (d,e). Cu-O bond accounts here for the possible presence of Cu-O, Cu-N and Cu-C bonds in the first coordination shell around Cu atom since they are practically indistinguishable with this method. Corrections to photoelectron reference energy ($\Delta E_0$) are shown in (c). The values of R-factor characterizing fit quality are shown in (f).

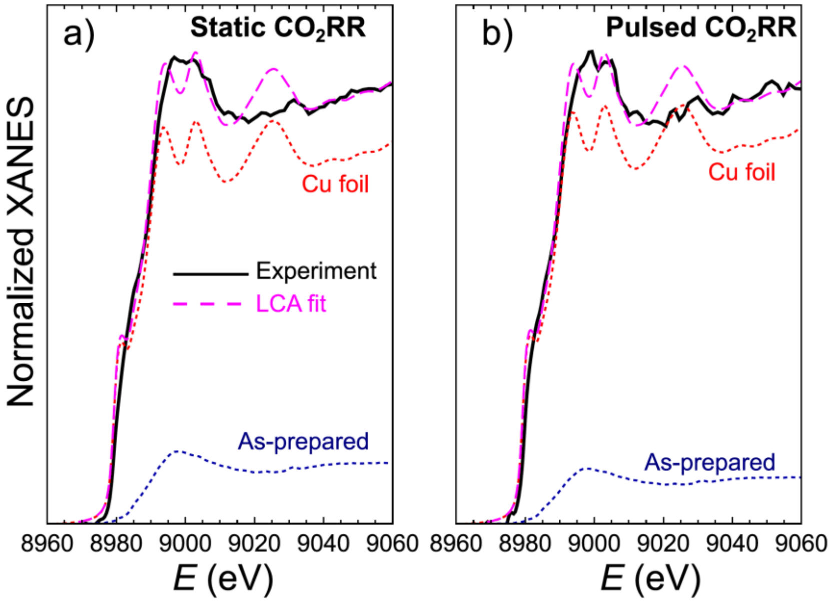


**Supplementary Figure 5. Representative examples of LCA-XANES fitting.** Experimental XANES spectrum (black solid line) and corresponding LCA-XANES fit (dashed magenta line) for the sample after 4000 s of static $CO_2$RR at -1.35 V (a) and after 4000 s of pulsed $CO_2$RR with $E_c$ = -1.35 V, $E_a$ = 0.44 V and $\Delta t_a = \Delta t_c$ = 30 s (b). Spectra for Cu foil and for the as-prepared Cu-N-C catalyst are used as references for LCA fit. The reference spectra are shown in both panels, scaled by their corresponding weights to the linear combination (red dotted line and blue dotted line, respectively)

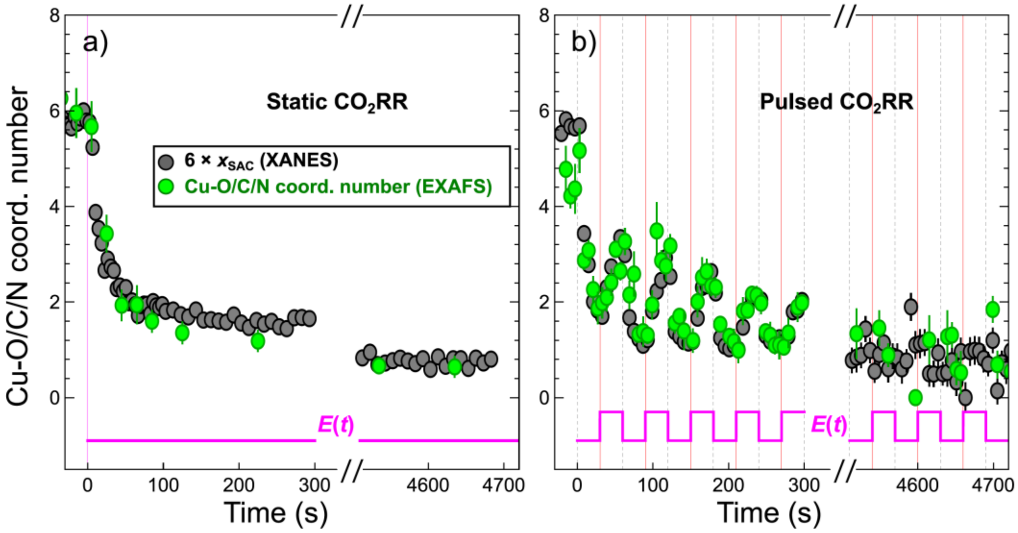


**Supplementary Figure 6. Comparison of XANES and EXAFS results for Cu-N-C under static and pulsed $CO_2$RR.** (a) Changes in the apparent Cu-O/N/C coordination number, as extracted from the analysis of *operando* Cu K-edge EXAFS data collected under static -1.35 V potential (a) and under pulsed $CO_2$RR with $E_c$ = -1.35 V, $E_a$ = 0.44 V and $\Delta t_a = \Delta t_c$ = 30 s (b). Corresponding changes in the concentration of singly dispersed Cu sites $x_{SAC}$, as obtained from LCA-XANES are also shown. LCA-XANES result is multiplied by 6 (the Cu-O/N/C coordination number for pure singly dispersed Cu sites), to facilitate the comparison between XANES and EXAFS results.

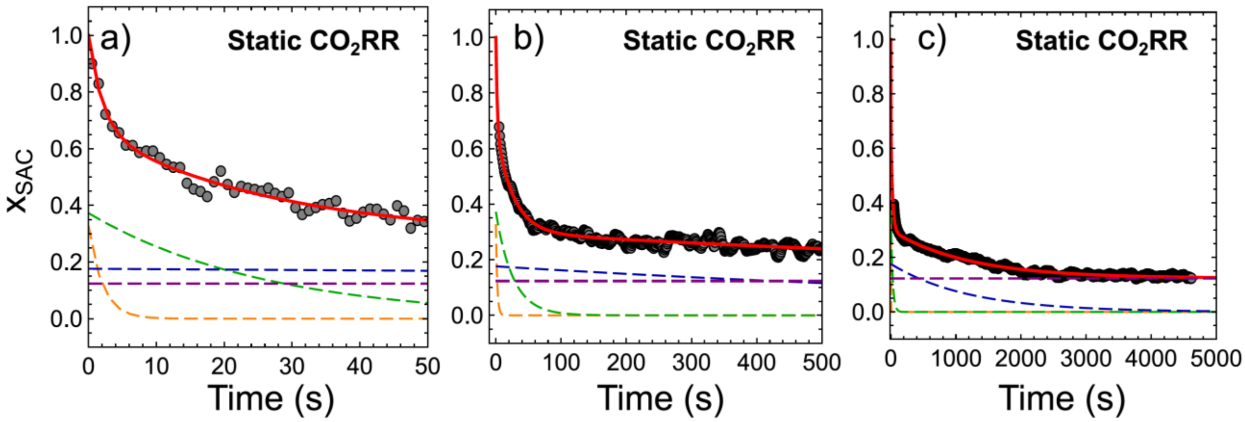


**Supplementary Figure 7. Changes in the concentration of singly dispersed Cu sites $x_{SAC}$, as extracted from the analysis of *operando* Cu K-edge XANES data collected under static -1.35 V potential.** Panels (a), (b) and (c) show the same data for different ranges of time. The time-dependency of $x_{SAC}$ can be fitted with a sum of exponential terms $\sum_{k=i}^{n} \mathrm{x_k} \exp(-t/\tau_k)$ (red solid line). We have found that at least four terms in the sum are necessary to properly describe the evolution of $x_{SAC}$ on different time scales, indicating that different singly dispersed Cu sites have different response to the applied potential. $x_1$= 33% of all singly dispersed sites are converted to metallic species very quickly, with corresponding $\tau_1$ = 2 s. The corresponding partial contribution of these species to the total concentration profile is indicated by the orange dashed line. $x_2$= 37% of all singly dispersed sites require an order of magnitude more time to be converted to metallic species, with corresponding $\tau_1$ = 26 s (green dashed line). $x_3$= 18% of all singly dispersed sites are converted to metal on even slower time scale, with corresponding $\tau_3$ = 1194 s (blue dashed line). Finally, $x_4$= 12% of all singly dispersed sites are not converted to metal at all (within the time of our experiment, purple dashed line).

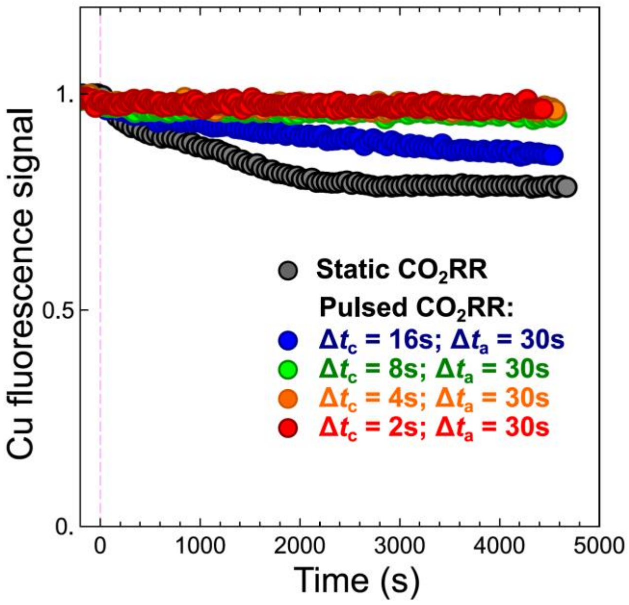


**Supplementary Figure 8. Relative changes in the Cu concentration during the static and pulsed $CO_2$RR.** Relative change in Cu fluorescence signal intensity as obtained under static $CO_2$RR at -1.35 V and pulsed $CO_2$RR experiments with $E_c$ = -1.35 V, $E_a$ = 0.44 V, $\Delta t_a$ = 30 s and different $\Delta t_c$ values.

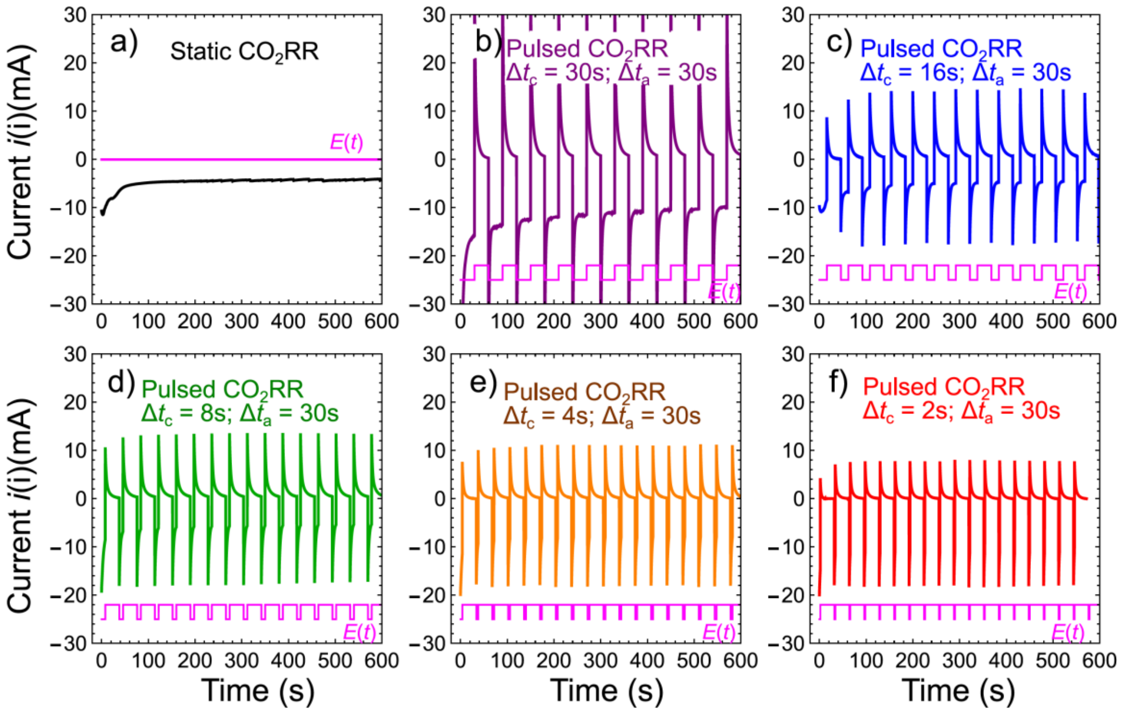


**Supplementary Figure 9. Time-dependencies of the current measured under static and pulsed $CO_2$RR.** Results obtained under static -1.35 V potential (a) and pulsed $CO_2$RR with $E_c$ = -1.35 V, $E_a$ = 0.44 V, $\Delta t_a$ = 30 s and different $\Delta t_c$ values (30 s (b), 16 s (c), 8 s (d), 4 s (e), 2 s (f)) are compared.

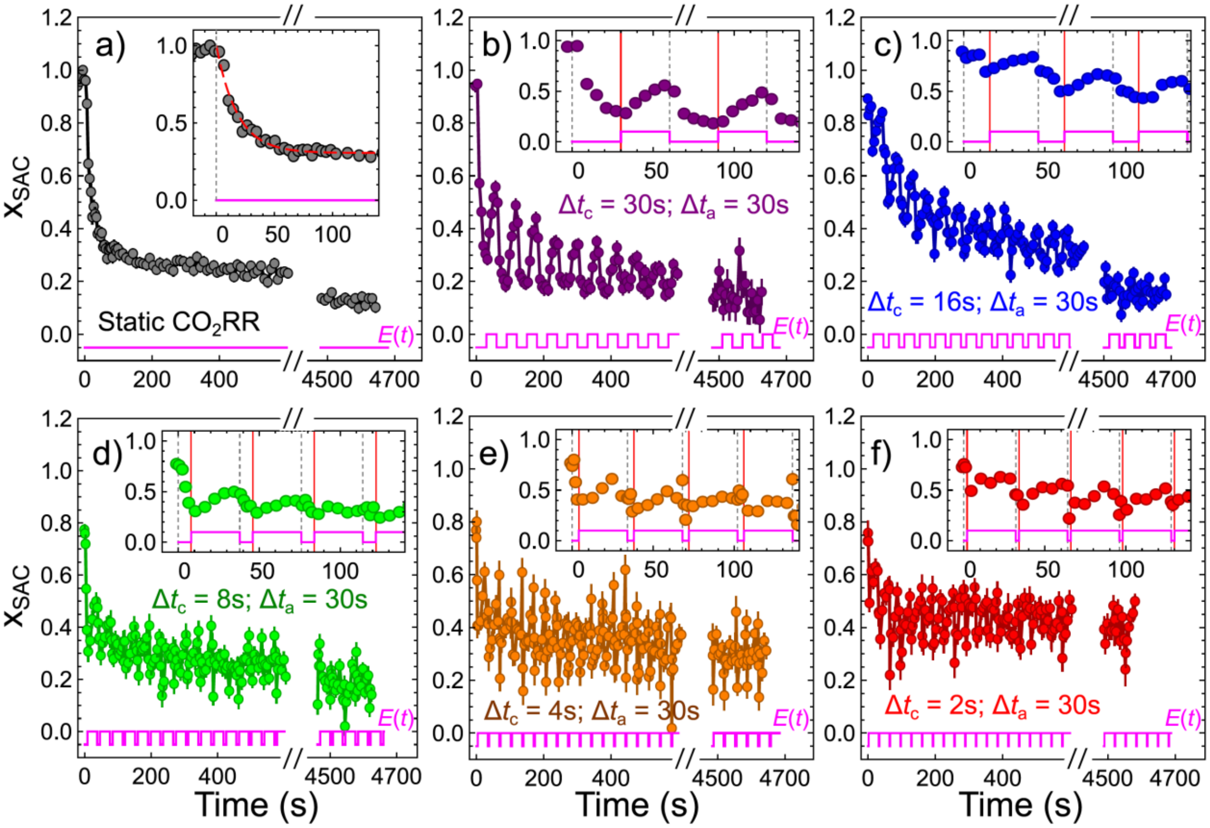


**Supplementary Figure 10. Changes in the concentration of singly dispersed Cu sites $x_{SAC}$ under static and pulsed $CO_2$RR with different $\Delta t_c$ values.** Changes in $x_{SAC}$, as extracted from the LCA of *operando* Cu K-edge XANES data collected under static -1.35 V potential (a) and pulsed $CO_2$RR with $E_c$ = -1.35 V, $E_a$ = 0.44 V, $\Delta t_a$ = 30 s and different $\Delta t_c$ values: 30 s (b), 16 s (c), 8 s (d), 4 s (e), 2 s (f). Insets show zoom-ins into the first 180 s of the experiment. The red dashed line in the inset in panel (a) – exponential fit of $x_{SAC}(t)$ values for the first 180 s.

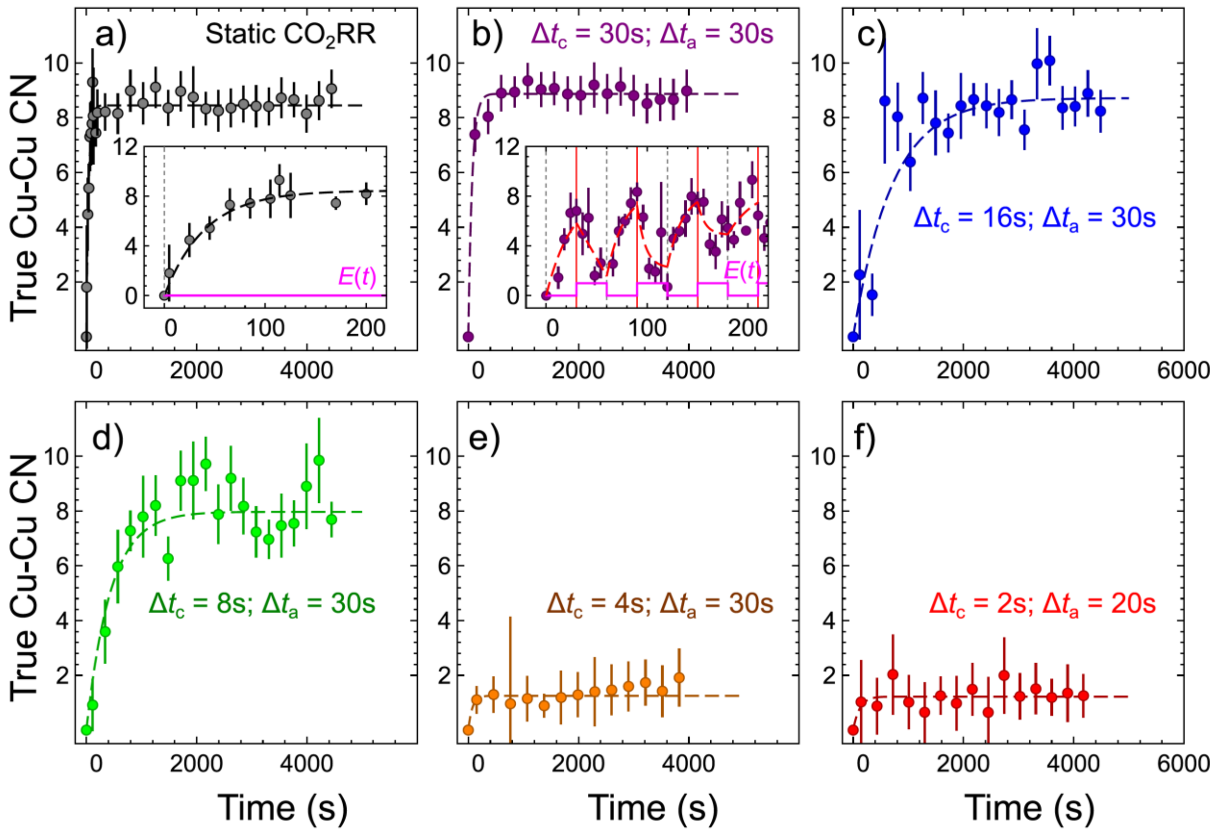


**Supplementary Figure 11. Changes in the true Cu-Cu coordination numbers under static and pulsed $CO_2$RR with different $\Delta t_c$ values.** True Cu-Cu coordination number, as extracted from the fits of *operando* Cu K-edge EXAFS data collected under (a) static -1.35 V potential (a) and pulsed $CO_2$RR with $E_c$ = -1.35 V, $E_a$ = 0.44 V, $\Delta t_a$ = 30 s and different $\Delta t_c$ values: 30 s (b), 16 s (c), 8 s (d), 4 s (e), 2 s (f). Insets in (a,b) show zoom-ins into the first 180 s of the corresponding experiment. Spectra used for EXAFS fitting were averaged over 228 s (a,d), 240 s (b), 230 s (c), 306 s (e) 288 s (f), 20-30 s (inset in (a)) or 6 s (inset in (b)). The depicted Cu-Cu coordination numbers are corrected for the averaging over metallic Cu and singly dispersed Cu sites, by dividing the apparent coordination numbers from EXAFS fit by (1-$x_{SAC}$). Black and red dashed lines in the insets – guides for the eye (exponential fits).

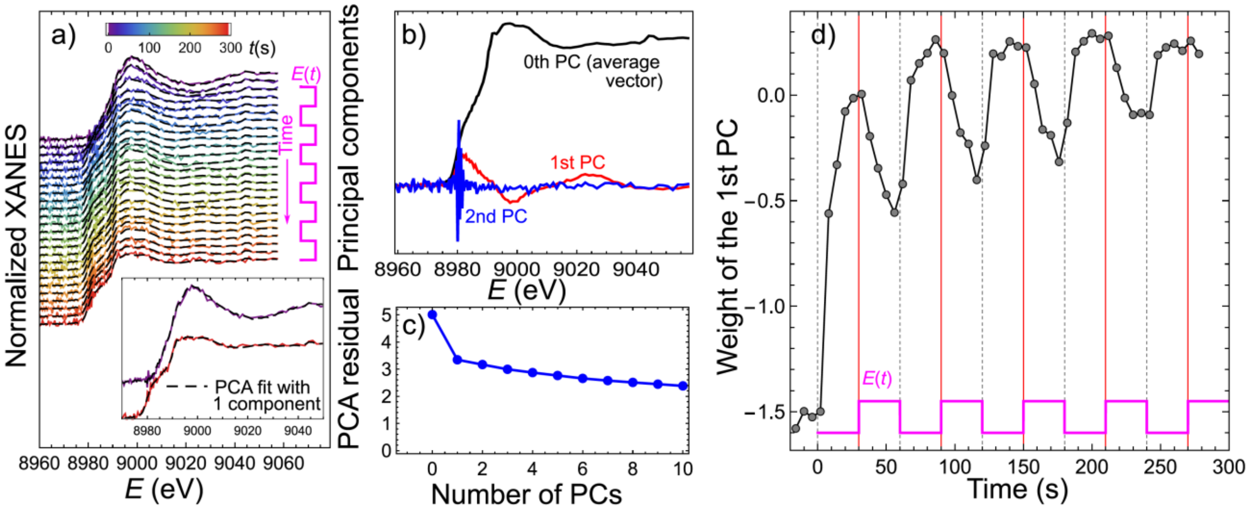


**Supplementary Figure 12. Principal component analysis for the *operando* XANES data for Cu-N-C catalyst.** Results obtained for the dataset collected under pulsed $CO_2$RR with $\Delta t_a = \Delta t_c = 30$s are shown. (a) Reconstruction of the spectra corresponding to the first potential pulse using the average spectrum and the 1st principal component. (b) Average spectrum and the first two principal components. (c) The total reconstruction error (residual) as a function of the number of PCs used. (d) Changes in the weight of the 1$^{st}$ PC during the first 300 s under pulsed $CO_2$RR.

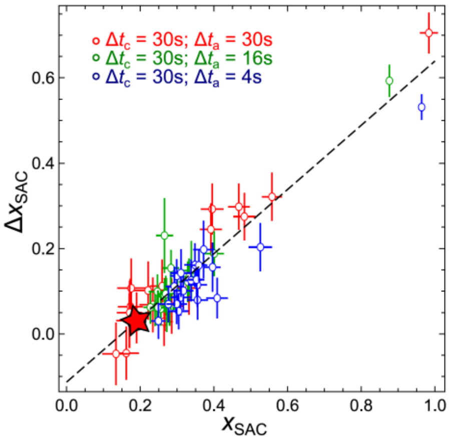


**Supplementary Figure 13. The relationship between the decrease in the concentration of singly dispersed Cu sites during each cathodic potential pulse and the concentration of these species at the onset of this pulse for 30 s cathodic pulses.** The decrease in the concentration of singly dispersed Cu sites ($\Delta x_{SAC}$) and the concentration of singly dispersed Cu sites at the onset of the respective cathodic potential pulse ($x_{SAC}$) are obtained from the analysis of experimental XANES data for pulsed $CO_2$RR with $E_c$ = -1.35 V, $E_a$ = 0.44 V, $\Delta t_c$ = 30 s and different $\Delta t_a$ values. Red, green and blue empty circles correspond to results obtained during the first 20 potential cycles (Figure 2b in the main text, Supplementary Figure 10). The red star marks the datapoint extracted from the averaged XANES data for the stationary state for $\Delta t_a = \Delta t_c$ = 30 s case (Figure 3a in the main text). Black dashed line – linear fit.

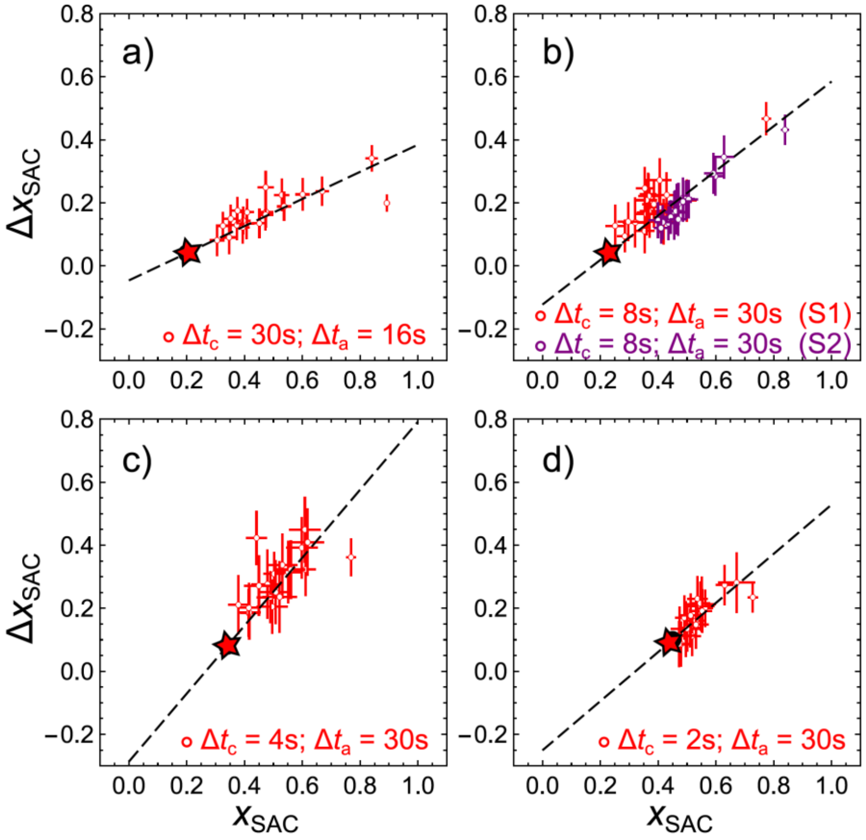


**Supplementary Figure 14. The relationship between the decrease in the concentration of singly dispersed Cu sites during each cathodic potential pulse and the concentration of these species at the onset of this pulse for cathodic pulses of different durations.** The decrease in the concentration of singly dispersed Cu sites during each cathodic potential pulse ($\Delta x_{SAC}$) and the concentration of singly dispersed Cu sites at the onset of the respective cathodic potential pulse ($x_{SAC}$) are obtained from the analysis of experimental XANES data for pulsed $CO_2$RR with $E_c$ = -1.35 V, $E_a$ = 0.44 V, $\Delta t_a$ = 30 s and different $\Delta t_c$ values: 16 s (a), 8 s (b), 4 s (c), 2 s (d). For $\Delta t_c$ = 8 s case, the results obtained in repeated measurements with two different samples are combined, to demonstrate the reproducibility of the results. Red and purple empty circles correspond to results obtained during the first 20 potential cycles (Supplementary Figure 10). The red stars mark the datapoints extracted from the averaged XANES data for the stationary state (Figure 3a in the main text). Black dashed lines – linear fits.

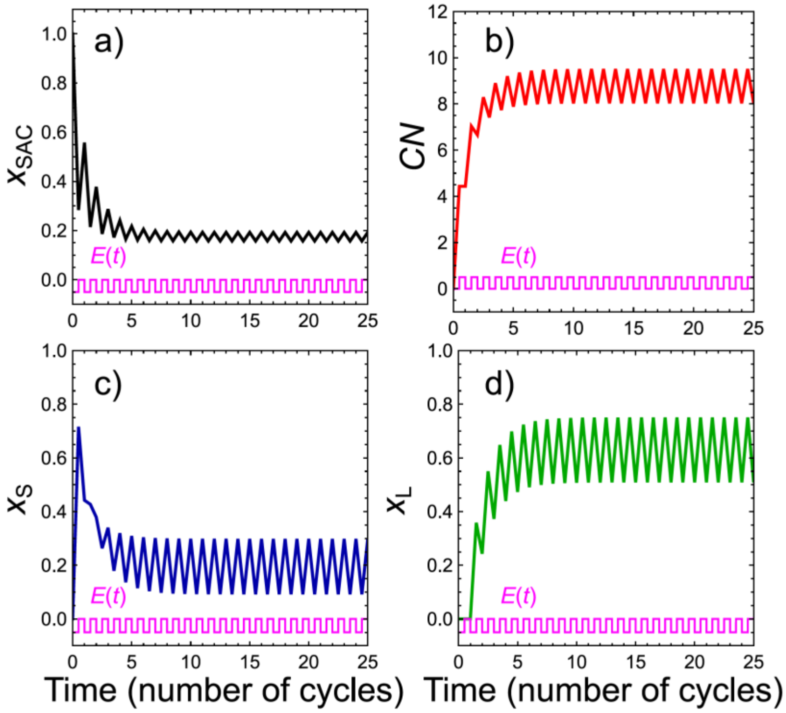


**Supplementary Figure 15**. **Application of the simple model, derived in Supplementary Note 4, to the pulsed $CO_2$RR with $\Delta t_a$ = $\Delta t_a$ = 30 s.** Changes in the concentration of singly dispersed Cu sites $x_{SAC}$ (a), average true Cu-Cu coordination number (b), concentrations of small Cu clusters (c) and large Cu particles (d) as a function of time.

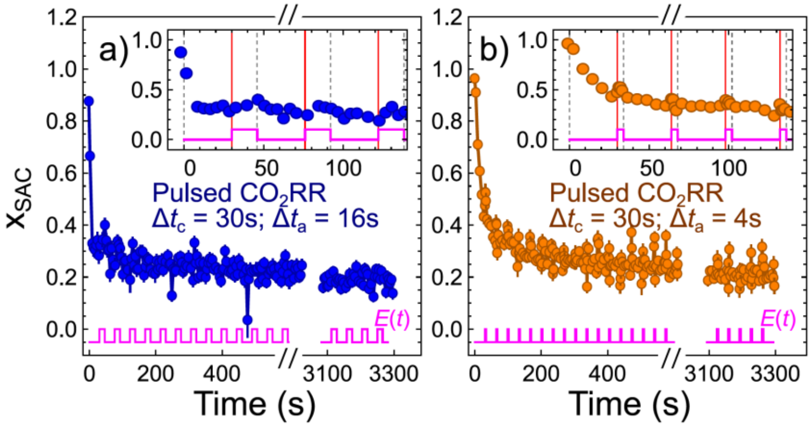


**Supplementary Figure 16. Effect of anodic pulse duration on the concentration of singly dispersed Cu species.** Changes in the concentration of singly dispersed Cu sites $x_{SAC}$, as extracted from the analysis of *operando* Cu K-edge XANES data collected under pulsed $CO_2$RR with $E_c$ = -1.35 V, $E_a$ = 0.44 V, $\Delta t_c$ = 30 s and different $\Delta t_a$ values: 16 s (a) and 4 s (b). Insets show zoom-ins into the first 180 s of the experiment.

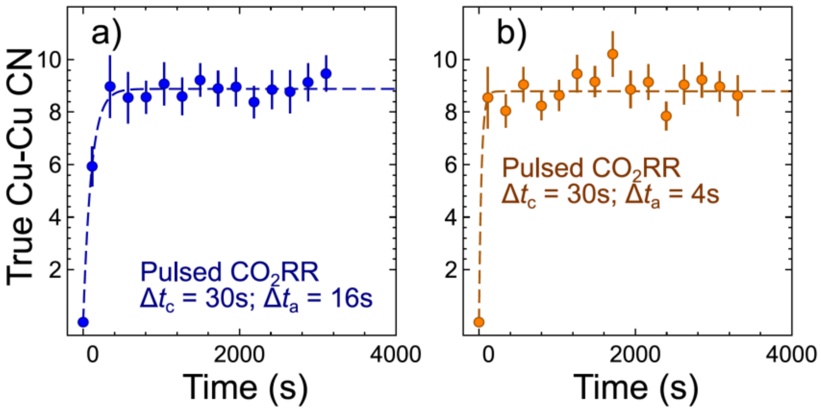


**Supplementary Figure 17. Effect of the anodic pulse duration on the Cu-Cu coordination numbers.** Changes in the Cu-Cu coordination number, as extracted from the analysis of *operando* Cu K-edge EXAFS data collected under pulsed $CO_2$RR with $E_c$ = -1.35 V, $E_a$ = 0.44 V, $\Delta t_c$ = 30 s and different $\Delta t_a$ values: 16 s (a) and 4 s (b). Spectra used for EXAFS fitting were averaged over 230 s (a) or 272 s (b). The depicted Cu-Cu coordination numbers are corrected for the averaging over metallic Cu and singly dispersed Cu sites, by dividing the apparent coordination numbers from EXAFS fit by (1-$x_{SAC}$).

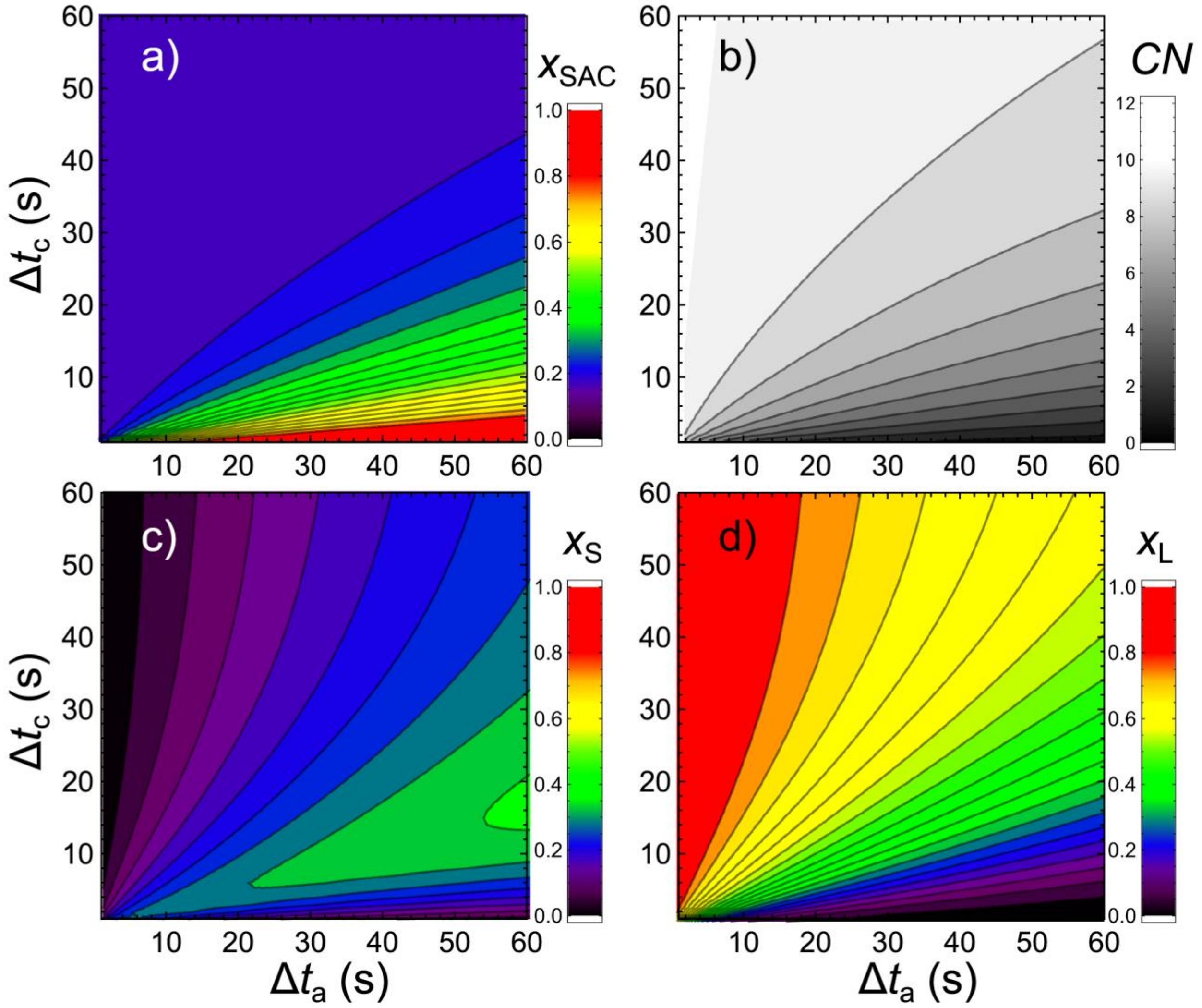


**Supplementary Figure 18. Catalyst structure parameters in the stationary state for different $\Delta t_a$ and $\Delta t_c$ values, as derived from our model, introduced in Supplementary Note 4.** (a) Average concentrations of singly dispersed cationic Cu. (b) Corresponding true average Cu-Cu coordination numbers. (c) Corresponding concentrations of small Cu clusters. (d) Corresponding concentrations of large Cu nanoparticles.

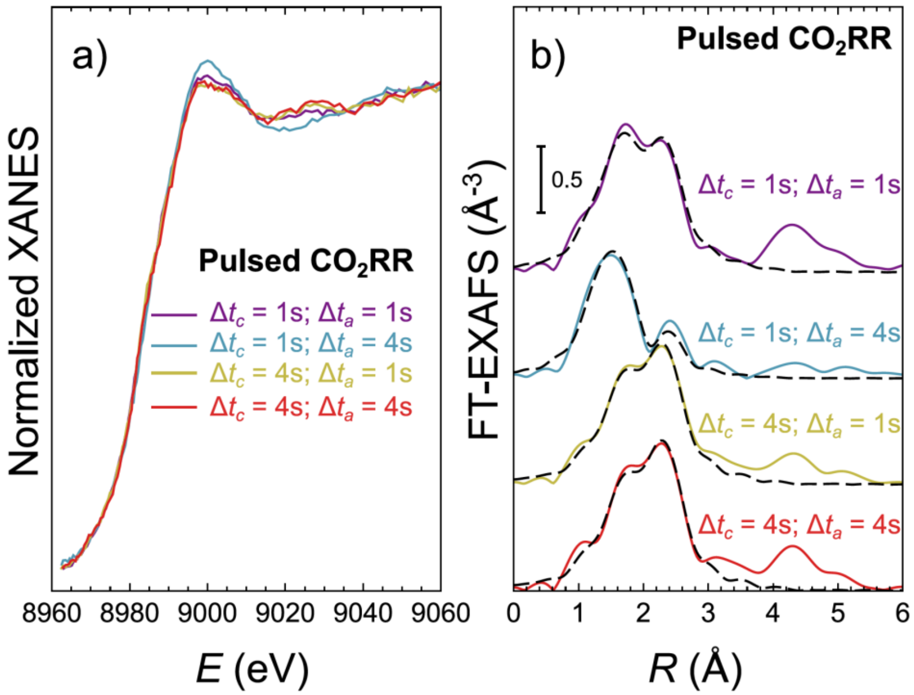


**Supplementary Figure 19. Additional *operando* XAS data collected under pulsed $CO_2$RR with different pulse durations.** Cu K-edge XANES (a) and Fourier-transformed (FT) EXAFS spectra for Cu-N-C catalysts collected in stationary state under pulsed $CO_2$RR with $E_c$ = -1.35 V, $E_a$ = 0.44 V, $\Delta t_a$ values 1 s or 4 s and $\Delta t_c$ values 1 s or 4 s. Black dashed lines in (b) – results of EXAFS data fitting. Spectra are collected at KMC3 beamline at BESSY II synchrotron, with an acquisition time of ca. 9 min per spectrum. Due to a technical issue with the beamline optics, the energy resolution in these spectra is lower than in other spectra discussed in this work. However, using reference spectra collected during the same beamtime for the LCA-XANES analysis and for the determination of $S_0^2$ factor in the EXAFS analysis, an accurate quantitative analysis of the average concentrations of cationic species and of the average Cu-Cu coordination numbers is still possible.

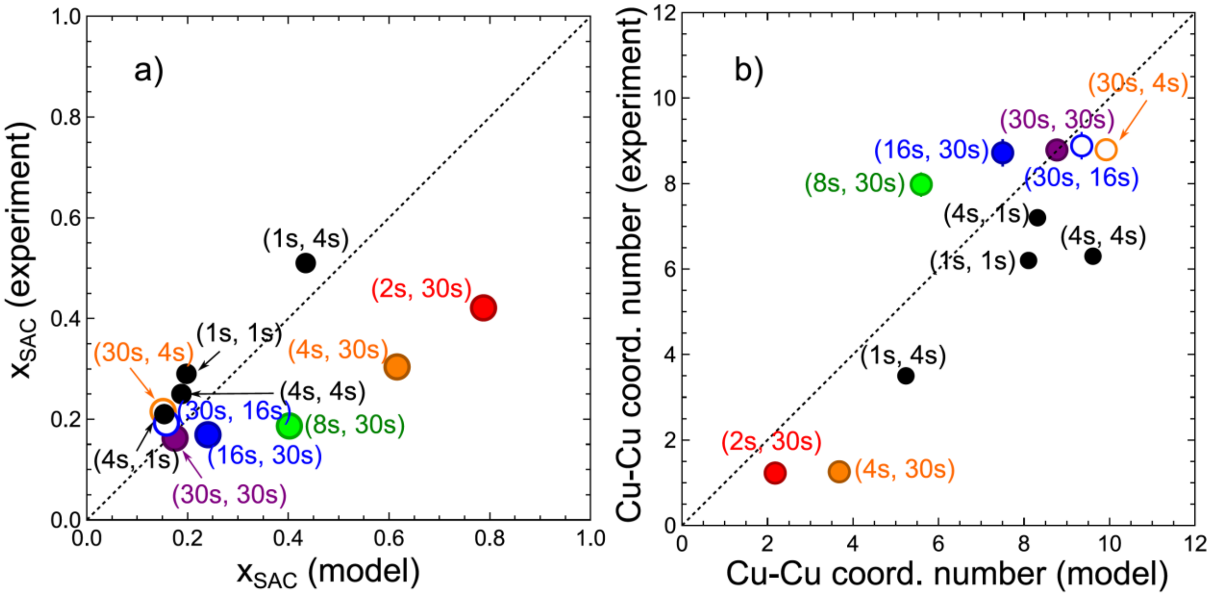


**Supplementary Figure 20. Validation of the model introduced in Supplementary Note 4.** (a) Average concentrations of singly dispersed cationic species, predicted by the model, are compared with the results of the experimental XAS data analysis. Filled large colored discs correspond to the experimental results with $\Delta t_a = 30$ s and varied $\Delta t_c$ (Figures 2 in the main text, Supplementary Figures 10-11), open large discs correspond to the experimental results with $\Delta t_c = 30$ s and varied $\Delta t_a$ (Supplementary Figures 13-14), black small discs correspond to the experimental results with $\Delta t_a$ values 1 s or 4 s and $\Delta t_c$ values 1 s or 4 s (Supplementary Figure 19). Corresponding durations of the applied pulses are indicated in the plot as ($\Delta t_a$, $\Delta t_c$). (b) Corresponding Cu-Cu coordination numbers.

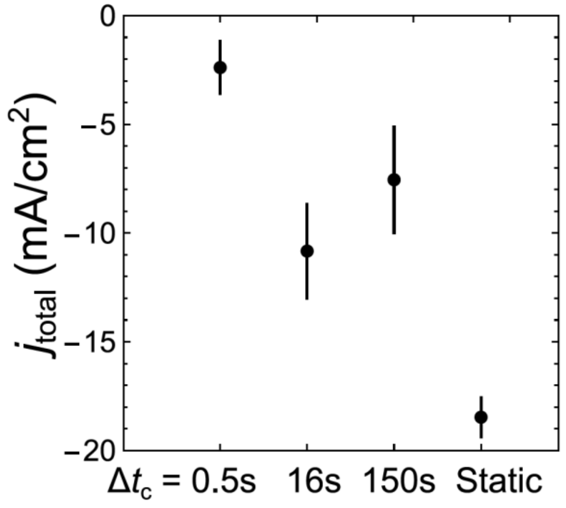


**Supplementary Figure 21. Total current density for static (at -1.35 V) and pulsed $CO_2$RR.** Results for $E_c$ = -1.35 V, $E_a$ = 0.44 V, $\Delta t_a$ =30 s and varied $\Delta t_c$ values are shown. Each measurement is performed for a fresh sample. Uncertainties are estimated by comparing the results of at least three repeated measurements using fresh samples.

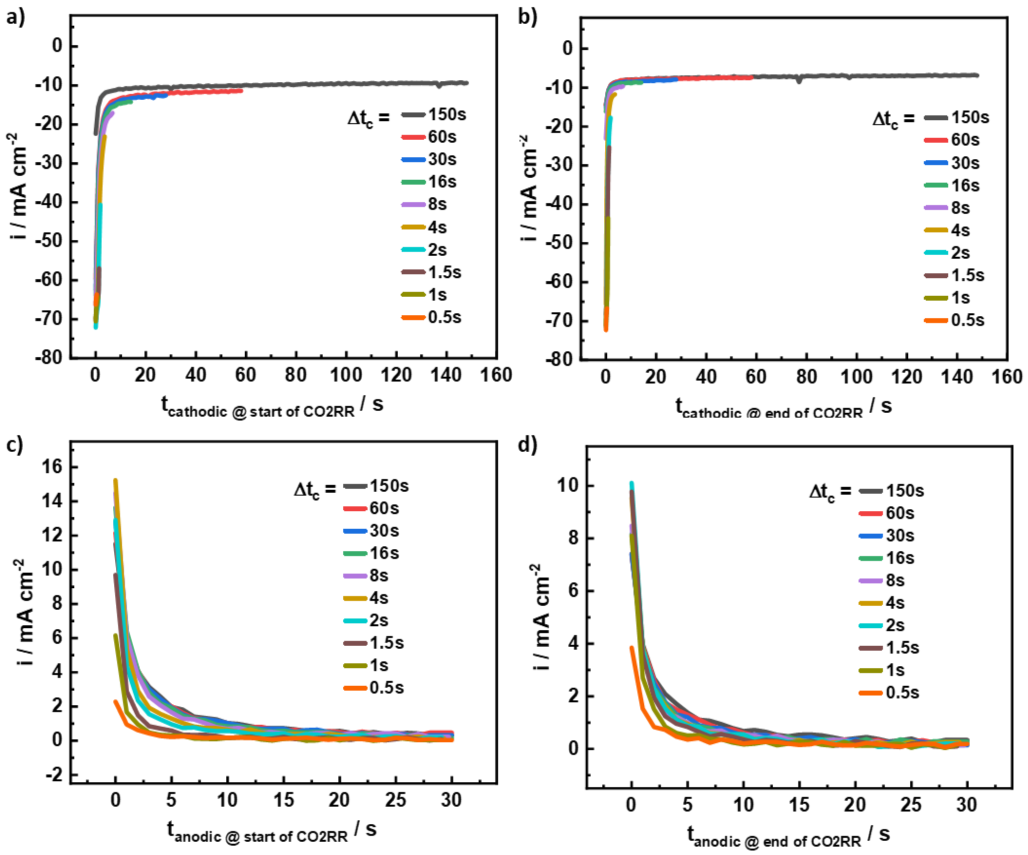


**Supplementary Figure 22. Pulsed current density profiles. Stacks of the cathodic (a,b) and anodic (c,d) pulsed current profiles at the beginning (a,c) and at the end of 4000 s $CO_2$RR for different cathodic pulse lengths.** For all pulses $E_c$ = -1.35 V, $E_a$ = 0.44 V and $\Delta t_a$ =30 s. Measurements are performed for the same sample, exposed to air for 30 min in between different pulse conditions. Note that the current values may differ from those in Supplementary Figure 9 due to the differences in the resistance for the single compartment cells used for the *operando* XAS measurements, and for the H-type cell employed for electrochemical characterization.

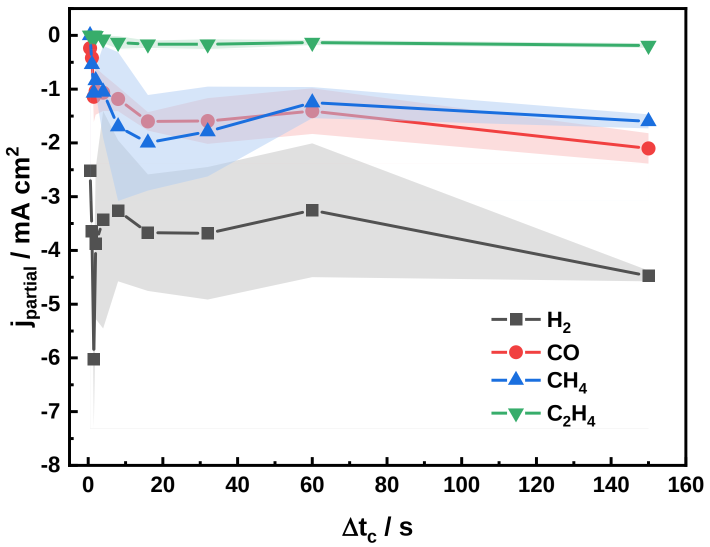


**Supplementary Figure 23. Pulsed partial current profiles.** Corresponding partial current densities for $H_2$ and the main gaseous $CO_2RR$ products, CO, $CH_4$ and $C_2H_4$ as a function of $\Delta t_c$. For all pulses $E_c$ = -1.35 V, $E_a$ = 0.44 V and $\Delta t_a$ =30 s. Measurements are performed for the same sample, exposed to air for 30 min in between different pulse conditions. Shaded areas indicate the standard deviation of individual measurements.

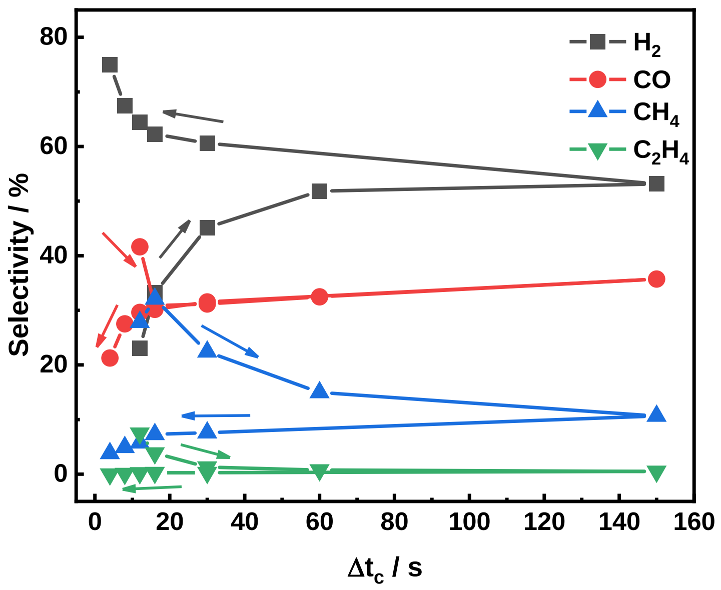


**Supplementary Figure 24. Sample history effect.** Corresponding selectivities for $H_2$ and the main gaseous $CO_2$RR products, CO, $CH_4$ and $C_2H_4$ as a function of $\Delta t_c$. For all pulses $E_c$ = -1.35 V, $E_a$ = 0.44 V and $\Delta t_a$ =30 s. The reported values are normalized assuming a FE of 100% for the detected products. A fresh sample is used for pulsed conditions with $\Delta t_c$ = 12 s, and then re-used, while, first, increasing towards $\Delta t_c$ until it reaches 150 s, and then reducing it until it reaches 0.5 s. In-between different pulse conditions, the sample is exposed to open circuit potential for 30 min.

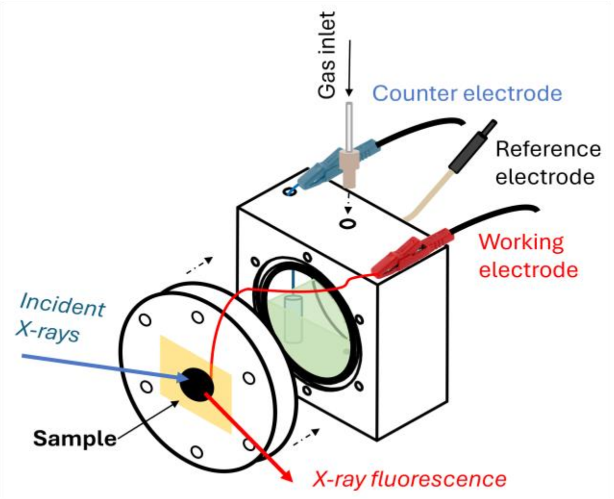


**Supplementary Figure 25. Schematical depiction of a single compartment electrochemical cell used for *operando* XAS measurements.** Adapted with permission from Ref.[6] Copyright (2021) American Chemical Society.

## Supplementary Tables

**Supplementary Table 1 | EXAFS data fitting results for the Cu-N-C catalysts under static -1.35 V potential.** The obtained values for Cu-Cu and Cu-O coordination numbers (*N*), bond length (*R*) and bond length disorder ($\sigma^2$). Cu-O bond accounts here for the possible presence of Cu-O, Cu-N and Cu-C bonds in the first coordination shell around Cu atom since they are practically indistinguishable with this method. Corrections to photoelectron reference energy ($\Delta E_0$) and the values of R-factor characterizing fit quality are also shown. Uncertainties of the last digit are shown in the parentheses.

| Time (s) | $N_{Cu-Cu}$ | $R_{Cu-Cu}$(Å) | $\sigma^2_{Cu-Cu}$(Å$^2$) | $N_{Cu-O}$ | $R_{Cu-O}$(Å) | $\sigma^2_{Cu-O}$(Å$^2$) | $\Delta E_0(eV)$ | R-factor (%) |
|---|---|---|---|---|---|---|---|---|
| -275 | - | - | - | 5.8(5) | 1.88(1) | 0.010(2) | -1(8) | 2.1 |
| -175 | - | - | - | 5.9(5) | 1.900(9) | 0.011(2) | -0(7) | 2.2 |
| -75 | - | - | - | 6.2(5) | 1.89(1) | 0.013(2) | -1(9) | 2.7 |
| 25 | 4(2) | 2.52(2) | 0.020(6) | 4.5(5) | 1.91(1) | 0.013(3) | 1.2(9) | 3.3 |
| 125 | 7.1(7) | 2.544(6) | 0.015(1) | 1.4(2) | 1.939(9) | 0.005(4) | 3.2(5) | 1.3 |
| 225 | 7.0(6) | 2.555(5) | 0.013(1) | 1.2(2) | 1.90(1) | 0.006(4) | 3.3(5) | 1 |
| 285 | 6.3(5) | 2.548(5) | 0.012(1) | 1.1(1) | 1.929(6) | 0.002(2) | 2.8(4) | 0.6 |
| 305 | 5.4(4) | 2.534(4) | 0.008(1) | 1.5(2) | 1.903(7) | 0.008(3) | 2.6(4) | 0.6 |
| 325 | 7.9(7) | 2.550(5) | 0.012(1) | 1.1(3) | 1.96(1) | 0.009(7) | 4.1(5) | 1 |
| 345 | 6.1(6) | 2.543(6) | 0.010(1) | 1.1(2) | 1.90(1) | 0.006(5) | 3.3(6) | 1.2 |
| 365 | 7.7(7) | 2.554(6) | 0.014(1) | 1.0(2) | 1.92(1) | 0.004(4) | 3.4(5) | 1.1 |
| 385 | 5.9(4) | 2.552(4) | 0.011(1) | 1.1(1) | 1.891(6) | 0.003(3) | 3.0(4) | 0.6 |
| 405 | 7.9(5) | 2.548(4) | 0.014(1) | 0.9(1) | 1.978(8) | 0.001(3) | 3.4(3) | 0.6 |
| 525 | 6.4(5) | 2.556(5) | 0.011(1) | 1.0(2) | 1.92(1) | 0.003(4) | 3.5(5) | 0.8 |
| 625 | 7.6(6) | 2.553(5) | 0.013(1) | 0.8(2) | 1.92(1) | 0.001(4) | 3.5(5) | 1 |
| 725 | 8.1(6) | 2.552(4) | 0.014(1) | 0.7(1) | 1.93(1) | 0.004(4) | 3.4(4) | 0.7 |
| 825 | 7.4(6) | 2.551(6) | 0.012(1) | 1.0(3) | 1.95(1) | 0.008(7) | 3.8(5) | 1.1 |
| 925 | 7.5(8) | 2.557(7) | 0.012(1) | 0.9(4) | 1.94(2) | 0.01(1) | 4.2(7) | 1.8 |
| 1025 | 7.4(5) | 2.553(3) | 0.012(1) | 0.8(1) | 1.920(9) | 0.003(3) | 3.4(3) | 0.6 |
| 1125 | 7.7(8) | 2.558(7) | 0.012(1) | 0.8(3) | 1.93(2) | 0.005(8) | 4.0(6) | 1.7 |
| 1225 | 8.4(6) | 2.550(5) | 0.013(1) | 0.8(2) | 1.94(1) | 0.004(6) | 3.5(4) | 0.9 |
| 1325 | 7.6(6) | 2.553(5) | 0.013(1) | 0.8(2) | 1.92(1) | 0.003(6) | 3.5(5) | 1.1 |
| 1425 | 7.4(5) | 2.555(4) | 0.011(1) | 0.7(1) | 1.897(9) | 0.004(4) | 3.5(4) | 0.5 |
| 1525 | 7.4(6) | 2.554(5) | 0.012(1) | 0.7(2) | 1.93(1) | 0.004(7) | 3.7(5) | 0.9 |
| 1625 | 7.6(5) | 2.551(5) | 0.012(1) | 0.7(2) | 1.96(2) | 0.008(8) | 3.7(4) | 0.8 |
| 1725 | 8.0(7) | 2.564(6) | 0.012(1) | 0.6(2) | 1.92(2) | 0.002(9) | 4.2(5) | 1.3 |
| 1825 | 8.2(6) | 2.556(5) | 0.012(1) | 0.7(3) | 1.95(2) | 0.007(9) | 3.9(5) | 1 |
| 1925 | 7.2(7) | 2.565(6) | 0.011(1) | 0.6(2) | 1.91(1) | 0.007(7) | 4.0(6) | 1.2 |
| 2025 | 8.2(8) | 2.565(6) | 0.012(1) | 0.5(2) | 1.91(2) | 0.008(8) | 4.3(6) | 1.4 |
| 2125 | 7.5(6) | 2.551(4) | 0.012(1) | 0.7(1) | 1.89(1) | 0.004(4) | 3.0(4) | 0.8 |
| 2225 | 6.6(4) | 2.554(4) | 0.009(1) | 0.8(1) | 1.880(8) | 0.003(3) | 2.9(4) | 0.5 |

| | | | | | | | | |
|---|---|---|---|---|---|---|---|---|
| 2325 | 7.4(7) | 2.559(6) | 0.010(1) | 0.6(2) | 1.91(2) | 0.008(8) | 4.0(6) | 1 |
| 2425 | 7.2(6) | 2.555(5) | 0.010(1) | 0.7(2) | 1.89(1) | 0.005(5) | 3.5(5) | 0.8 |
| 2525 | 8.4(7) | 2.560(5) | 0.013(1) | 0.7(3) | 1.96(2) | 0.01(1) | 3.9(5) | 1.1 |
| 2625 | 7.5(7) | 2.560(6) | 0.011(1) | 0.6(2) | 1.91(2) | 0.00(1) | 4.0(6) | 1.4 |
| 2725 | 7.8(6) | 2.555(5) | 0.012(1) | 0.6(2) | 1.91(1) | 0.006(6) | 3.5(4) | 0.8 |
| 2825 | 7.8(7) | 2.556(6) | 0.012(1) | 0.6(2) | 1.92(2) | 0.001(8) | 3.7(6) | 1.3 |
| 2925 | 7.1(4) | 2.551(3) | 0.010(1) | 0.7(1) | 1.895(8) | 0.002(4) | 2.9(3) | 0.3 |
| 3025 | 8.1(8) | 2.558(6) | 0.012(1) | 0.5(2) | 1.90(2) | 0.009(9) | 3.9(6) | 1.4 |
| 3125 | 8.0(7) | 2.563(5) | 0.012(1) | 0.4(2) | 1.89(2) | 0.008(8) | 4.1(5) | 1.3 |
| 3225 | 7.6(5) | 2.560(4) | 0.011(1) | 0.5(1) | 1.91(1) | 0.006(6) | 3.6(4) | 0.7 |
| 3325 | 8.0(6) | 2.562(5) | 0.011(1) | 0.5(2) | 1.91(2) | 0.001(8) | 4.1(5) | 0.9 |
| 3425 | 7.9(5) | 2.556(4) | 0.011(1) | 0.6(1) | 1.91(1) | 0.005(5) | 3.6(4) | 0.7 |
| 3525 | 6.9(6) | 2.552(5) | 0.010(1) | 0.8(2) | 1.92(1) | 0.005(7) | 3.5(5) | 1 |
| 3625 | 7.8(6) | 2.553(4) | 0.011(1) | 0.6(2) | 1.91(1) | 0.001(6) | 3.5(4) | 0.7 |
| 3725 | 7.9(6) | 2.554(5) | 0.012(1) | 0.7(3) | 1.96(2) | 0.01(1) | 3.8(5) | 0.9 |
| 3825 | 8.6(7) | 2.558(5) | 0.013(1) | 0.5(2) | 1.94(2) | 0.008(8) | 3.8(5) | 0.9 |
| 3925 | 7.1(5) | 2.556(4) | 0.010(1) | 0.6(1) | 1.89(1) | 0.005(5) | 3.5(4) | 0.8 |
| 4025 | 7.1(7) | 2.566(6) | 0.010(1) | 0.4(2) | 1.91(3) | 0.01(1) | 4.5(6) | 1.4 |
| 4125 | 8.1(5) | 2.554(3) | 0.012(1) | 0.5(1) | 1.92(1) | 0.005(5) | 3.3(3) | 0.5 |
| 4225 | 8.3(6) | 2.559(4) | 0.012(1) | 0.5(1) | 1.91(2) | 0.005(5) | 3.6(5) | 0.8 |
| 4325 | 8.1(6) | 2.557(5) | 0.011(1) | 0.6(3) | 1.97(3) | 0.01(1) | 3.9(5) | 1 |
| 4425 | 7.7(6) | 2.558(5) | 0.012(1) | 0.6(2) | 1.90(2) | 0.006(6) | 3.9(5) | 1 |
| 4525 | 8.4(6) | 2.549(4) | 0.013(1) | 0.7(1) | 1.92(1) | 0.005(5) | 3.1(4) | 0.7 |
| 4625 | 7.8(5) | 2.556(5) | 0.011(1) | 0.6(2) | 1.94(2) | 0.006(8) | 3.6(5) | 0.8 |

**Supplementary Table 2 | EXAFS data fitting results for the Cu-N-C catalysts under pulsed $CO_2$RR with $E_c$ = -1.35 V, $E_a$ = 0.44 V, $\Delta t_a$ = 30 s and $\Delta t_c$ =30 s.** The obtained values for Cu-Cu and Cu-O coordination numbers (*N*), bond length (*R*) and bond length disorder ($\sigma^2$). Cu-O bond accounts here for the possible presence of Cu-O, Cu-N and Cu-C bonds in the first coordination shell around Cu atom since they are practically indistinguishable with this method. Corrections to photoelectron reference energy ($\Delta E_0$) and the values of R-factor characterizing fit quality are also shown. Uncertainties of the last digit are shown in the parentheses.

| Time (s) | $N_{Cu-Cu}$ | $R_{Cu-Cu}$(Å) | $\sigma^2_{Cu-Cu}$(Å$^2$) | $N_{Cu-O}$ | $R_{Cu-O}$(Å) | $\sigma^2_{Cu-O}$(Å$^2$) | $\Delta E_0(eV)$ | R-factor (%) |
|---|---|---|---|---|---|---|---|---|
| 120 | 4.9(4) | 2.525(4) | 0.009(1) | 2.0(2) | 1.933(7) | 0.009(2) | 1.7(5) | 0.5 |
| 360 | 6.0(4) | 2.533(4) | 0.008(1) | 1.5(2) | 1.941(8) | 0.008(4) | 2.4(5) | 0.6 |
| 600 | 7.3(5) | 2.538(4) | 0.009(1) | 1.1(2) | 1.94(1) | 0.004(5) | 2.7(5) | 0.6 |
| 840 | 7.7(5) | 2.537(4) | 0.009(1) | 0.8(2) | 1.94(1) | 0.004(4) | 2.7(4) | 0.4 |
| 1080 | 8.0(5) | 2.538(4) | 0.009(1) | 0.8(2) | 1.94(1) | 0.001(5) | 3.0(4) | 0.5 |
| 1320 | 7.7(5) | 2.533(4) | 0.008(1) | 0.9(2) | 1.94(1) | 0.002(5) | 2.5(5) | 0.5 |
| 1560 | 7.7(5) | 2.533(4) | 0.008(1) | 0.9(2) | 1.96(1) | 0.002(5) | 2.3(4) | 0.4 |
| 1800 | 7.5(5) | 2.532(4) | 0.008(1) | 0.9(2) | 1.95(1) | 0.001(5) | 2.2(5) | 0.5 |
| 2040 | 7.4(6) | 2.536(5) | 0.008(1) | 0.9(2) | 1.95(1) | 0.001(5) | 2.4(5) | 0.6 |
| 2280 | 7.5(6) | 2.534(5) | 0.008(1) | 1.1(3) | 1.95(1) | 0.005(6) | 2.5(6) | 0.8 |
| 2520 | 7.3(6) | 2.531(5) | 0.007(1) | 1.1(4) | 1.96(2) | 0.01(1) | 2.2(6) | 0.8 |
| 2760 | 7.8(6) | 2.533(4) | 0.007(1) | 0.9(2) | 1.96(1) | 0.005(5) | 2.9(5) | 0.6 |
| 3000 | 7.3(6) | 2.537(5) | 0.008(1) | 1.0(3) | 1.96(1) | 0.004(6) | 2.6(6) | 0.8 |
| 3240 | 6.9(5) | 2.530(5) | 0.008(1) | 1.2(3) | 1.94(1) | 0.006(6) | 2.0(6) | 0.7 |
| 3480 | 7.1(6) | 2.535(5) | 0.008(1) | 1.1(3) | 1.95(1) | 0.003(6) | 2.6(6) | 0.9 |
| 3720 | 7.2(6) | 2.527(5) | 0.007(1) | 1.0(3) | 1.95(1) | 0.002(6) | 1.9(6) | 0.7 |
| 3960 | 7.0(6) | 2.528(5) | 0.007(1) | 1.3(4) | 1.95(1) | 0.009(7) | 2.0(6) | 0.8 |
| 4200 | 6.4(5) | 2.530(5) | 0.006(1) | 1.0(2) | 1.95(1) | 0.002(5) | 1.7(6) | 0.6 |
| 4440 | 6.6(6) | 2.528(6) | 0.006(1) | 1.2(4) | 1.97(2) | 0.009(8) | 2.1(7) | 0.9 |

**Supplementary Table 3 | EXAFS data fitting results for the Cu-N-C catalysts under pulsed $CO_2$RR with $E_c$ = -1.35 V, $E_a$ = 0.44 V, $\Delta t_a$ = 30 s and $\Delta t_c$ =16 s.** The obtained values for Cu-Cu and Cu-O coordination numbers (*N*), bond length (*R*) and bond length disorder ($\sigma^2$). Cu-O bond accounts here for the possible presence of Cu-O, Cu-N and Cu-C bonds in the first coordination shell around Cu atom since they are practically indistinguishable with this method. Corrections to photoelectron reference energy ($\Delta E_0$) and the values of R-factor characterizing fit quality are also shown. Uncertainties of the last digit are shown in the parentheses.

| Time (s) | $N_{Cu-Cu}$ | $R_{Cu-Cu}$(Å) | $\sigma^2_{Cu-Cu}$(Å$^2$) | $N_{Cu-O}$ | $R_{Cu-O}$(Å) | $\sigma^2_{Cu-O}$(Å$^2$) | $\Delta E_0(eV)$ | R-factor (%) |
|---|---|---|---|---|---|---|---|---|
| 115 | 0.5(6) | 2.60(2) | 0.01(1) | 4.5(5) | 1.87(1) | 0.010(2) | -2(1) | 2.9 |
| 345 | 0.9(4) | 2.55(1) | 0.005(5) | 2.5(3) | 1.86(1) | 0.006(3) | -2(1) | 2.5 |
| 575 | 5(1) | 2.56(2) | 0.016(4) | 2.4(7) | 1.93(2) | 0.017(8) | 3(1) | 7.1 |
| 805 | 6.1(9) | 2.55(1) | 0.016(2) | 1.4(3) | 1.91(1) | 0.006(5) | 2.7(8) | 2.9 |
| 1035 | 4.8(8) | 2.553(9) | 0.009(2) | 1.5(3) | 1.89(1) | 0.007(5) | 2.6(9) | 2.5 |
| 1265 | 7.1(7) | 2.547(7) | 0.015(1) | 1.1(2) | 1.93(1) | 0.004(4) | 3.0(6) | 1.6 |
| 1495 | 7(1) | 2.56(1) | 0.012(2) | 0.7(3) | 1.90(2) | 0.01(1) | 4(1) | 3.2 |
| 1725 | 6.4(6) | 2.557(5) | 0.010(1) | 0.8(1) | 1.88(1) | 0.004(4) | 3.5(5) | 1 |
| 1955 | 8(1) | 2.560(6) | 0.013(2) | 0.7(1) | 1.89(2) | 0.006(6) | 3.9(6) | 1.7 |
| 2185 | 7.6(5) | 2.552(4) | 0.012(1) | 0.7(1) | 1.908(9) | 0.004(4) | 3.3(4) | 0.6 |
| 2415 | 7.4(7) | 2.554(5) | 0.012(1) | 0.7(2) | 1.90(1) | 0.005(5) | 3.7(5) | 1.2 |
| 2645 | 7.2(7) | 2.550(6) | 0.011(1) | 0.7(2) | 1.91(2) | 0.001(6) | 3.8(6) | 1.4 |
| 2875 | 7.6(6) | 2.549(5) | 0.012(1) | 0.7(2) | 1.91(1) | 0.005(5) | 3.3(5) | 0.8 |
| 3105 | 6.6(6) | 2.548(5) | 0.009(1) | 0.8(2) | 1.87(1) | 0.005(5) | 3.3(5) | 1 |
| 3565 | 9.2(8) | 2.549(6) | 0.013(1) | 0.5(3) | 1.98(3) | 0.01(1) | 4.0(5) | 1.2 |
| 3795 | 7.4(7) | 2.552(4) | 0.010(1) | 0.7(2) | 1.87(1) | 0.005(5) | 3.5(5) | 1 |
| 4025 | 7.5(6) | 2.557(5) | 0.011(1) | 0.7(2) | 1.89(1) | 0.006(6) | 3.7(5) | 1 |
| 4255 | 8.1(7) | 2.557(5) | 0.012(1) | 0.5(2) | 1.89(2) | 0.008(8) | 3.9(7) | 1.3 |
| 4485 | 7.5(7) | 2.561(5) | 0.010(1) | 0.6(2) | 1.89(2) | 0.007(7) | 4.1(5) | 1 |

**Supplementary Table 4 | EXAFS data fitting results for the Cu-N-C catalysts under pulsed $CO_2$RR with $E_c$ = -1.35 V, $E_a$ = 0.44 V, $\Delta t_a$ = 30 s and $\Delta t_c$ =8 s.** The obtained values for Cu-Cu and Cu-O coordination numbers (*N*), bond length (*R*) and bond length disorder ($\sigma^2$). Cu-O bond accounts here for the possible presence of Cu-O, Cu-N and Cu-C bonds in the first coordination shell around Cu atom since they are practically indistinguishable with this method. Corrections to photoelectron reference energy ($\Delta E_0$) and the values of R-factor characterizing fit quality are also shown. Uncertainties of the last digit are shown in the parentheses.

| Time (s) | $N_{Cu-Cu}$ | $R_{Cu-Cu}$(Å) | $\sigma^2_{Cu-Cu}$(Å$^2$) | $N_{Cu-O}$ | $R_{Cu-O}$(Å) | $\sigma^2_{Cu-O}$(Å$^2$) | $\Delta E_0(eV)$ | R-factor (%) |
|---|---|---|---|---|---|---|---|---|
| 152 | 0.5(5) | 2.56(2) | 0.01(1) | 2.5(4) | 1.87(2) | 0.006(4) | -3(1) | 4.4 |
| 456 | 2.6(8) | 2.55(1) | 0.007(4) | 1.7(3) | 1.87(1) | 0.002(3) | 1(1) | 3.5 |
| 760 | 4.2(9) | 2.56(1) | 0.011(3) | 1.8(4) | 1.90(1) | 0.008(5) | 2(1) | 3.6 |
| 1064 | 5.4(5) | 2.548(6) | 0.015(1) | 1.6(2) | 1.899(6) | 0.006(2) | 1.7(5) | 0.8 |
| 1368 | 6(1) | 2.57(1) | 0.014(3) | 1.2(3) | 1.90(2) | 0.004(6) | 4(1) | 4 |
| 1672 | 6.8(9) | 2.560(8) | 0.015(2) | 1.0(2) | 1.89(1) | 0.001(4) | 2.9(7) | 2.1 |
| 1976 | 5.1(6) | 2.554(6) | 0.010(2) | 1.1(2) | 1.878(9) | 0.001(3) | 2.4(7) | 1.4 |
| 2280 | 7.4(8) | 2.566(8) | 0.016(2) | 1.2(3) | 1.94(1) | 0.008(6) | 3.5(6) | 2.1 |
| 2584 | 8(1) | 2.564(9) | 0.016(2) | 0.8(3) | 1.88(2) | 0.007(7) | 3.7(8) | 3.6 |
| 2888 | 8.3(8) | 2.547(7) | 0.016(1) | 0.9(3) | 1.94(1) | 0.005(7) | 3.4(6) | 1.7 |
| 3192 | 6.8(9) | 2.557(8) | 0.013(2) | 0.9(2) | 1.88(1) | 0.005(5) | 3.3(7) | 2.3 |
| 3496 | 7.3(9) | 2.552(8) | 0.014(2) | 1.2(4) | 1.97(2) | 0.01(1) | 3.6(7) | 2.5 |
| 3800 | 7.1(9) | 2.566(8) | 0.013(2) | 0.8(2) | 1.90(2) | 0.005(5) | 3.9(7) | 2.3 |
| 4104 | 6.1(7) | 2.554(7) | 0.012(2) | 1.0(2) | 1.89(1) | 0.004(4) | 3.2(7) | 1.8 |
| 4408 | 5.7(6) | 2.545(5) | 0.010(1) | 1.1(2) | 1.876(9) | 0.002(3) | 2.6(6) | 1 |
| 4712 | 6(1) | 2.566(8) | 0.012(2) | 0.8(3) | 1.89(2) | 0.006(6) | 4.0(9) | 3.2 |
| 5016 | 6.4(7) | 2.563(6) | 0.011(1) | 0.9(2) | 1.87(1) | 0.004(4) | 3.2(6) | 1.4 |
| 5320 | 8(1) | 2.57(1) | 0.014(2) | 0.5(3) | 1.90(3) | 0.01(1) | 4(1) | 5 |
| 5624 | 8(1) | 2.56(1) | 0.015(2) | 0.9(5) | 1.97(3) | 0.01(1) | 4.3(9) | 3.9 |
| 5928 | 6.4(5) | 2.552(5) | 0.012(1) | 1.0(1) | 1.902(8) | 0.003(3) | 2.9(5) | 0.8 |

**Supplementary Table 5 | EXAFS data fitting results for the Cu-N-C catalysts under pulsed $CO_2$RR with $E_c$ = -1.35 V, $E_a$ = 0.44 V, $\Delta t_a$ = 30 s and $\Delta t_c$ =4 s.** The obtained values for Cu-Cu and Cu-O coordination numbers ($N$), bond length ($R$) and bond length disorder ($\sigma^2$). Cu-O bond accounts here for the possible presence of Cu-O, Cu-N and Cu-C bonds in the first coordination shell around Cu atom since they are practically indistinguishable with this method. Corrections to photoelectron reference energy ($\Delta E_0$) and the values of R-factor characterizing fit quality are also shown. Uncertainties of the last digit are shown in the parentheses.

| Time (s) | $N_{Cu-Cu}$ | $R_{Cu-Cu}$(Å) | $\sigma^2_{Cu-Cu}$(Å$^2$) | $N_{Cu-O}$ | $R_{Cu-O}$(Å) | $\sigma^2_{Cu-O}$(Å$^2$) | $\Delta E_0(eV)$ | R-factor (%) |
|---|---|---|---|---|---|---|---|---|
| 153 | 0.6(2) | 2.58(2) | 0.005(5) | 3.0(4) | 1.85(2) | 0.006(3) | -4(1) | 3.8 |
| 459 | 0.6(3) | 2.56(1) | 0.005(5) | 3.0(2) | 1.838(8) | 0.007(2) | -2(7) | 1.1 |
| 1683 | 0.7(6) | 2.56(2) | 0.009(9) | 2.4(3) | 1.86(2) | 0.005(3) | -2(1) | 4.5 |
| 1989 | 0.8(5) | 2.53(2) | 0.007(7) | 2.3(3) | 1.86(1) | 0.005(3) | -2(1) | 3.7 |
| 2601 | 0.9(5) | 2.53(2) | 0.007(7) | 2.3(4) | 1.86(2) | 0.006(4) | -2(2) | 4.7 |
| 2907 | 1.0(5) | 2.55(2) | 0.006(6) | 2.3(4) | 1.87(2) | 0.006(4) | -2(2) | 4.7 |
| 3213 | 1.1(5) | 2.54(1) | 0.005(5) | 2.1(3) | 1.85(2) | 0.003(3) | -2(2) | 3.8 |

**Supplementary Table 6 | EXAFS data fitting results for the Cu-N-C catalysts under pulsed $CO_2$RR with $E_c$ = -1.35 V, $E_a$ = 0.44 V, $\Delta t_a$ = 30 s and $\Delta t_c$ =2 s.** The obtained values for Cu-Cu and Cu-O coordination numbers ($N$), bond length ($R$) and bond length disorder ($\sigma^2$). Cu-O bond accounts here for the possible presence of Cu-O, Cu-N and Cu-C bonds in the first coordination shell around Cu atom since they are practically indistinguishable with this method. Corrections to photoelectron reference energy ($\Delta E_0$) and the values of R-factor characterizing fit quality are also shown. Uncertainties of the last digit are shown in the parentheses.

| Time (s) | $N_{Cu-Cu}$ | $R_{Cu-Cu}$(Å) | $\sigma^2_{Cu-Cu}$(Å$^2$) | $N_{Cu-O}$ | $R_{Cu-O}$(Å) | $\sigma^2_{Cu-O}$(Å$^2$) | $\Delta E_0(eV)$ | R-factor (%) |
|---|---|---|---|---|---|---|---|---|
| 144 | 0.4(7) | 2.63(2) | 0.02(2) | 3.4(4) | 1.85(1) | 0.008(2) | -3(1) | 2.7 |
| 432 | 0.4(4) | 2.60(2) | 0.01(1) | 3.4(3) | 1.86(1) | 0.008(2) | -3(1) | 2.6 |
| 720 | 0.8(6) | 2.61(2) | 0.008(8) | 3.5(5) | 1.85(2) | 0.009(3) | -4(2) | 4.2 |
| 1008 | 0.5(5) | 2.62(3) | 0.01(1) | 3.1(2) | 1.857(7) | 0.006(1) | -2(7) | 1 |
| 1296 | 0.3(5) | 2.66(5) | 0.01(1) | 3.0(3) | 1.87(2) | 0.006(2) | -2(1) | 1.2 |
| 1584 | 0.6(3) | 2.66(1) | 0.006(6) | 3.1(2) | 1.862(7) | 0.006(1) | -1(6) | 1 |
| 1872 | 0.5(4) | 2.61(2) | 0.01(1) | 3.2(4) | 1.84(1) | 0.007(3) | -4(1) | 3 |
| 2160 | 0.7(4) | 2.61(2) | 0.007(7) | 3.4(3) | 1.86(1) | 0.009(2) | -2(9) | 2 |
| 2736 | 0.7(5) | 2.62(2) | 0.008(8) | 3.8(5) | 1.85(1) | 0.012(3) | -3(1) | 3.3 |
| 3024 | 0.6(4) | 2.62(4) | 0.01(1) | 3.2(6) | 1.84(1) | 0.008(5) | -4(1) | 2.8 |
| 3312 | 0.7(4) | 2.61(1) | 0.007(7) | 3.1(2) | 1.86(1) | 0.006(2) | -2(9) | 1.5 |
| 3600 | 0.6(3) | 2.62(1) | 0.006(6) | 3.0(2) | 1.853(9) | 0.005(2) | -2(8) | 1.3 |
| 3888 | 0.7(5) | 2.62(2) | 0.008(8) | 3.1(4) | 1.84(2) | 0.007(3) | -4(1) | 3.4 |
| 4176 | 0.7(4) | 2.62(1) | 0.006(6) | 2.9(3) | 1.84(1) | 0.005(2) | -4(1) | 2.1 |

**Supplementary Table 7 | Faradaic Efficiencies (FE) and partial current densities (PCD) for static (at -1.35 V vs RHE) and pulsed $CO_2$RR.** Results for $E_c$ = -1.35 V, $E_a$ = 0.44 V, $\Delta t_a$ =30 s and varied $\Delta t_c$ values are shown. Each measurement is performed for a fresh sample. Uncertainties are estimated by comparing the results of at least three repeated measurements using fresh samples.

| **$\Delta t_c$** | **0.5s** | | **16s** | | **150s** | | **Static** | |
|---|---|---|---|---|---|---|---|---|
| | FE (%) | PCD (mA/cm$^2$) | FE (%) | PCD (mA/cm$^2$) | FE (%) | PCD (mA/cm$^2$) | FE (%) | PCD (mA/cm$^2$) |
| **$H_2$** | 86± 3 | -2± 1 | 31± 8 | -3± 1 | 46± 1 | -3.5± 0.2 | 47± 10 | -9± 1 |
| **CO** | 9± 3 | -0.2± 0.1 | 23± 5 | -2.5± -0.7 | 37± 1 | -2.8± 0.1 | 22± 4 | -4.5± 0.7 |
| **$CH_4$** | 0 | 0 | 25± 5 | -2.6± 0.5 | 5± 1 | -0.4± 0.1 | 10± 8 | -2± 2 |
| **$C_2H_4$** | 0 | 0 | 3± 2 | -0.3± 0.1 | 1.1± 0.3 | -0.09± 0.03 | 2± 1 | -0.4± 0.3 |
| **Acetaldehyde** | 0 | 0 | 2± 1 | -0.1± 0.1 | 1.3± 0.2 | -0.073± 0.004 | 1.9± 0.2 | -0.26± 0.04 |
| **Propion-aldehyde** | 0 | 0 | 0 | 0 | 0 | 0 | 0.03± 0.01 | -0.005± 0.002 |
| **EtOH** | 0 | 0 | 1.2± 0.6 | -0.09± 0.04 | 0.9± 0.2 | -0.06± 0.03 | 4± 1 | -0.7± 0.3 |
| **1-PrOH** | 0 | 0 | 0.05± 0.07 | -0.003± 0.004 | 0.26± 0.06 | -0.015± 0.002 | 1.4± 0.2 | -0.2± 0.05 |
| **Allyl alcohol** | 0 | 0 | 0.2± 0.2 | -0.01± 0.01 | 0.40± 0.08 | -0.023± 0.001 | 0.67± 0.05 | -0.09± 0.01 |
| **$HCOO^-$** | 2.5± 0.6 | -0.06± 0.04 | 14± 3 | -1.8± 0.2 | 5.9± 0.6 | -0.56± 0.05 | 8± 2 | -1.8± 0.6 |
| **Acetate** | 0 | 0 | 0.66± 0.06 | -0.09± 0.02 | 0.11± 0.02 | -0.011± 0.04 | 0.4± 0.3 | -0.1± 0.09 |

## Supplementary References


1 Martini, A. *et al.* Tracking the evolution of single atom catalysts for the CO2 electrocatalytic reduction using operando X-ray absorption spectroscopy and machine learning. *J. Am. Chem. Soc.* **145**, 17351-17366 (2023).

2 Hursán, D. *et al.* Reversible evolution of metal-nitrogen-doped carbon catalysts during $CO_2$ electroreduction: operando X-ray absorption spectroscopy study. *Advanced Materials* **36**, 2307809 (2023).

3 Calvin, S. *et al.* Determination of crystallite size in a magnetic nanocomposite using extended x-ray absorption fine structure. *Journal of Applied Physics* **94**, 778-783 (2003).

4 Timoshenko, J. *et al.* Subnanometer substructures in nanoassemblies formed from clusters under reactive atmosphere revealed using machine learning. *Journal of Physical Chemistry C* **122**, 21686-21693 (2018).

5 Timoshenko, J. *et al.* Linking the evolution of catalytic properties and structural changes in copper–zinc nanocatalysts using operando EXAFS and neural-networks. *Chemical Science* **11**, 3727-3736 (2020).

6 Timoshenko, J. & Roldan Cuenya, B. In Situ/Operando Electrocatalyst Characterization by X-ray Absorption Spectroscopy. *Chemical Reviews* **121**, 882–961 (2021).

7 Timoshenko, J. & Frenkel, A. I. “Inverting” X-ray absorption spectra of catalysts by machine learning in search for activity descriptors. *ACS Catalysis* **9**, 10192-10211 (2019).

8 Martini, A. & Borfecchia, E. Spectral Decomposition of X-ray Absorption Spectroscopy Datasets: Methods and Applications. *Crystals* **10**, 664 (2020).

9 Timoshenko, J. *et al.* Steering the structure and selectivity of CO2 electroreduction by potential pulses. *Nature Catalysis* **5**, 259-267 (2022).
